# Nano-Enhanced Phase Reinforced Magnesium Matrix Composites: A Review of the Matrix, Reinforcement, Interface Design, Properties and Potential Applications

**DOI:** 10.3390/ma17102454

**Published:** 2024-05-19

**Authors:** Jiao-Yi Ren, Guan-Cheng Ji, Hao-Rui Guo, Yu-Meng Zhou, Xin Tan, Wen-Fang Zheng, Qian Xing, Jia-Yi Zhang, Jing-Ran Sun, Hong-Yu Yang, Feng Qiu, Qi-Chuan Jiang

**Affiliations:** Key Laboratory of Automobile Materials, Ministry of Education and Department of Materials Science and Engineering, Jilin University, Renmin Street No. 5988, Changchun 130025, China; renjy1620@mails.jlu.edu.cn (J.-Y.R.); jigc1620@mails.jlu.edu.cn (G.-C.J.); guohr1620@mails.jlu.edu.cn (H.-R.G.); zhouym1620@mails.jlu.edu.cn (Y.-M.Z.); tanxin1621@mails.jlu.edu.cn (X.T.); zhengwf1620@mails.jlu.edu.cn (W.-F.Z.); xingqian1621@mails.jlu.edu.cn (Q.X.); zhangjy1621@mails.jlu.edu.cn (J.-Y.Z.); sjr22@mails.jlu.edu.cn (J.-R.S.); yanghongyu2021@jlu.edu.cn (H.-Y.Y.); jqc@jlu.edu.cn (Q.-C.J.)

**Keywords:** magnesium matrix composites, nano-enhanced phase, ceramic nanoparticles, interfacial design, properties and applications

## Abstract

Magnesium matrix composites are essential lightweight metal matrix composites, following aluminum matrix composites, with outstanding application prospects in automotive, aerospace lightweight and biomedical materials because of their high specific strength, low density and specific stiffness, good casting performance and rich resources. However, the inherent low plasticity and poor fatigue resistance of magnesium hamper its further application to a certain extent. Many researchers have tried many strengthening methods to improve the properties of magnesium alloys, while the relationship between wear resistance and plasticity still needs to be further improved. The nanoparticles added exhibit a good strengthening effect, especially the ceramic nanoparticles. Nanoparticle-reinforced magnesium matrix composites not only exhibit a high impact toughness, but also maintain the high strength and wear resistance of ceramic materials, effectively balancing the restriction between the strength and toughness. Therefore, this work aims to provide a review of the state of the art of research on the matrix, reinforcement, design, properties and potential applications of nano-reinforced phase-reinforced magnesium matrix composites (especially ceramic nanoparticle-reinforced ones). The conventional and potential matrices for the fabrication of magnesium matrix composites are introduced. The classification and influence of ceramic reinforcements are assessed, and the factors influencing interface bonding strength between reinforcements and matrix, regulation and design, performance and application are analyzed. Finally, the scope of future research in this field is discussed.

## 1. Introduction

Magnesium, owing to its excellent properties like good castability, low density and abundant reserves on earth in the structural materials has the potential to replace existing used metals and alloys such as steel and aluminum in terms of annual metal production in the world [1,2]. Meanwhile, the potential applications of Magnesium Matrix Composites (MMCs) in new energy vehicles, biomedical materials and hydrogen storage have attracted worldwide attention [1,3,4,5,6,7,8]. Magnesium alloys with good thermal conductivity and high specific strength, excellent stiffness and damping capabilities, as well as outstanding machinability and favorable recyclability, are attractive for structural components in the automotive and aerospace industries [9,10,11,12,13,14,15]. The magnesium matrix composites with excellent mechanical properties have greatly pushed the development of magnesium matrix composites. For example, the presence of aluminum leads to the increase of 180 MPa in the strength of a magnesium–aluminum alloy [4].

However, as a structural material, its inherent low modulus of elasticity, low plasticity, fatigue resistance and poor corrosion resistance limit its extensive use in critical engineering applications [5,6,13,14,15]. In recent decades, many strengthening methods such as precipitation strengthening, solid-solution hardening, work hardening and grain-size refinement have been used to control the comprehensive properties of MMCs. Although these traditional methods have played a certain positive role, they also bring undeniable negative impacts. For example, solid-solution hardening and grain-size refinement have a significant influence on the density and cost of Mg alloys due to the increased content of alloying elements [3,4,5]. For the near interface crack of the bi-material interface, the work hardening overmatching (Δ*n* < 0; n is the work hardening exponent of the material, Δ*n* is the difference between the work hardening exponents of the two materials) also has slight detrimental effects on fracture resistance [11]. Considerable efforts have been made to produce magnesium matrix composites reinforced with nano-phases, such as ceramic particles. These composites not only have high impact toughness, but also possess the high strength and wear resistance of ceramic materials, which overcome these shortcomings [10].

Up to now, many scholars have reviewed magnesium matrix composites, but most of them summarize a certain characteristic. In the introduction of different magnesium materials, Prasad et al. [4] comprehensively introduced the information of magnesium alloys and their composites, especially the classification of magnesium alloys, the influence of elements on their mechanical properties, and the manufacturing technology and corrosion of magnesium alloys, but the introduction of magnesium matrix composites is less common. The research of Saikrishna et al. [3] focused on the reinforcement of carbon nanotubes, and our work made up for the lack of richness in the introduction of reinforcements, to a certain extent. Guan et al. [5] focused on the design, process and performance of magnesium matrix composites. This article only focuses on the discussion of different types of reinforcements, and the content of interface optimization is limited. Our work introduces, in detail, the effect of interface regulation on the properties of magnesium matrix composites. For performance and application, other studies pay more attention to a certain aspect of performance research. For example, Saikrishna et al. [3] focused on the hardness and corrosion properties of magnesium matrix composites, Tang et al. [10] focused on wear resistance, etc., and this paper more comprehensively and systematically describes most of the properties of MMCs, and can be combined with practical applications to enable readers to understand the performance of MMCs more intuitively.

Therefore, it is particularly important to summarize the research results in related fields more comprehensively. This work aims to review the research progress of nano-reinforced magnesium matrix composites in a more comprehensive way. From the perspective of the matrix type, nano-reinforced material (especially ceramic nano-reinforced material) type, interface control and design, performance, and application, the theory and case are combined to enable readers to better understand the development of magnesium matrix composites, in order to provide some help for the exploration of this field.

## 2. Research and Development of Matrix in Magnesium Matrix Composites

Magnesium alloys have inevitable disadvantages, such as poor strength and corrosion resistance. Among the magnesium-based materials, magnesium matrix composites possess extremely outstanding properties [16,17,18]. Nowadays, owing to the sustainable requirements of energy saving and emission reduction, it has attracted extensive attention around the world. A suitable matrix is the lower limit of the properties of MMCs. The selection of the matrix of MMCs influences the mechanical properties in terms of wettability between matrix and reinforcement, especially for specific properties. Several promising magnesium matrices are introduced below.

### 2.1. Mg-Al-Zn (AZ) Matrix Composites

**Figure 1:**
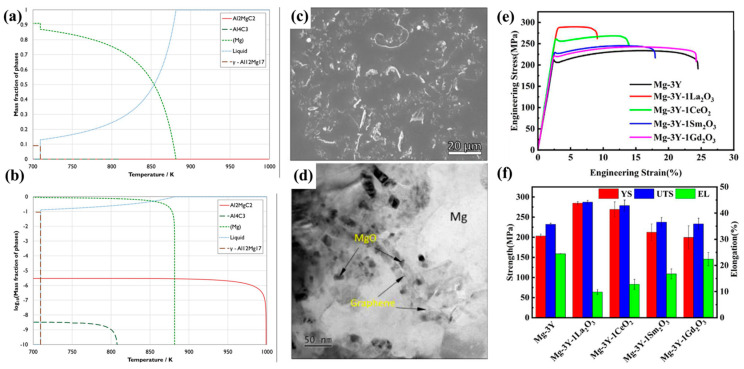
The 91Mg-9Al wt% alloy was heated to 1000 k and modified by 0.5wt% C. The phase (**a**) linear and (**b**) logarithmic mass fraction images of the Scheil solidification model were obtained [25]. SEM morphologies of (**c**) Mg_6_Zn and (**d**) ZnO-0.5GO/Mg_6_Zn [27]. Comparison of tensile stress–strain (**e**) and strength of Mg3Y alloy and Mg_3_Y-REmOn composites (**f**) [29].

At present, AZ magnesium alloys have been widely used as the magnesium matrix in MMCs [19,20,21,22,23,24]. With the increase in aluminum content, the strength, hardness and castability of magnesium matrix increase, while the plasticity and corrosion resistance decrease. The plasticity of the matrix increases with increasing zinc content, while it will decrease when the content is higher than 2wt%. Therefore, the content of elements in the matrix needs to be strictly controlled. For carbon-reinforced MMCs, the Al_2_MgC_2_ phase is formed along the grain boundary with the increase in Al content. Deffrennes et al. [25] obtained a 70Mg-19Al-11Cwt% sample in a Ta crucible and quenched at 1273 K for 240 h to obtain Al_2_MgC_2_ crystal. A relatively accurate ternary system model was obtained by comparing the decomposition temperatures calculated by differential thermal analysis (DTA) and discrete Fourier transform (DFT). Figure 1a gives the phase diagram obtained by Gulliver solidification simulation, indicating that the content of γ-Al_12_Mg_17_ is very small and disappears completely after 720 °C (Figure 1b). It is proved that Al_2_MgC_2_ is the only stable solid phases in the Al-Mg-C ternary system at higher temperatures. Thermodynamic calculation results confirm that Al_2_MgC_2_ is an inoculant for grain refinement in a Mg-Al alloy. In coated carbon fiber-reinforced MMCs, excellent nucleation agents can be obtained by adjusting the element content in the matrix, adding reinforcements, and appropriate interface cutting to obtain an eminent magnesium matrix.

In situ reaction synthesis is a commonly used method for manufacturing composite materials. This method is based on the chemical reaction between matrix elements or between elements and compounds to produce reinforcements in the metal matrix. Compared to conventional ex situ composites, in situ MMCs have a stronger interface bonding, regular atomic arrangement and fine and uniformly distributed grains. Therefore, the appropriate selection and control of matrix elements provide another idea for the synthesis of reinforcements and coatings.

For example, Xu et al. [26] prepared GO-SnO_2_/AZ31 matrix composites using SnO_2_ as graphene oxide (GO) coating by stirring casting and hot rolling process. They realized the excellent interface design between AZ31 matrix and reinforcement by using multi-layer graphene oxide and in situ synthesis. There is an interface that exists between particles of GO agglomerate and Mg matrix in GO/AZ31. Transmission electron microscopy analysis indicates that MgO nanoparticles were uniformly distributed on the base of GO-SnO_2_/AZ31. The MgO particles were formed by in situ synthesis of magnesium matrix and SnO_2_ coating. The large MgO particles and GO in GO-SnO_2_/AZ31 are uniformly dispersed, and the diameter of the nano-reinforced phase is about 0.21 nm. The in situ synthesis of MgO particles accompanied by the formation of Sn atom at the same time, and the dislocations are easier to accumulate, forming high-density stacking fault (SF). The mechanical properties of GO-SnO_2_/AZ31 composites (YS = 263 MPa, UTS = 308 MPa) are better than those of GO/AZ31 and AZ31 matrix. The SF interface improves the strain hardening ability of the composite, and the GO reinforcement enhances the electromagnetic interference shielding performance, leading to the excellent comprehensive performance.

Wang et al. [27] prepared nano-ZnO modified GO ZnO-0.5GO/Mg_6_Zn composites by the stirring casting method. The MgO interface was successfully synthesized in situ in a ZnO-Mg system. As shown in Figure 1c, graphite is uniformly dispersed on the matrix, and no agglomeration occurs. Figure 1d clearly shows that the MgO nanoparticles are distributed on the surface of the matrix containing graphene. The MgO nanoparticles have a strong interfacial bonding force, which is beneficial to the growth of graphene in the matrix. The formation of a lubricating layer caused by GO grown on the matrix reduces the wear of the matrix, thereby reducing the O_2_ generated by the friction loss. The wear mechanisms transform from oxidation wear to abrasive wear, and thus leads to the improvement in the hardness and wear resistance of the matrix. The in situ synthesis of new particles through the selection of coating and matrix improves the interface bonding and the regular arrangement of the matrix and the reinforcement, optimizing the performance of the composite. Compared with the above-mentioned GO-reinforced magnesium-based MMCs, the growth and distribution of GO on the matrix are different from substrates and in situ synthesis methods. GO becomes agglomerated into GO/AZ31, while it becomes dispersed in several other composites. The interface distribution of nano-reinforced phase particles in ZnO-0.5GO/Mg_6_Zn is better than that of GO-SnO_2_/AZ31. The choice of matrix has a remarkable effect on the growth of nano-reinforced particles on the surface. The same reinforcing phase exhibits different characteristics in different matrices.

AZ series magnesium alloys with the properties of low density and high elastic modulus limit their further application in the industrial production of magnesium alloys because of the characteristics of corrosion. In previous years, the industrial production of high-performance AZ magnesium alloy was realized by optimizing the element types of the matrix and improving the processing method of the matrix and the coating on the surface of the alloy. However, there are few studies on adding reinforcements and coating methods. Fortunately, magnesium matrix composites have obtained more excellent mechanical and wear resistance properties under various environmental conditions through microscopic interface design and mutual cooperation between the matrix, reinforcement and coating, which proves that it is of great potential to optimize matrix properties by designing and optimizing composite materials.

### 2.2. Research and Development of Mg-RE (WE) Matrix Composites

The addition of the Re element can refine the grains of α-Mg matrix, and thus leading to more excellent mechanical properties. The Re element can cooperate with the reinforcement (3D carbon nanotubes, ceramics, etc.) to improve the thermal conductivity because of its very low solubility in the magnesium. It is widely used in aerospace, electronics, energy storage and other realms [4]. The application of rare earth elements is unbalanced, and rare earth elements are also very expensive. The reserves of La and Ce are tremendous. For example, the adding of the La element (3~5 wt%) to magnesium alloy leads to the formation of Mg_12_La (Mg_12_Ce) phase, which makes the corrosion rate faster [28]. Liang Ren et al. [29] prepared Mg_3_Y-RE_m_O_n_ in situ reinforced MMCs by redox reaction of RE_m_O_n_ and Y. The UTS of Mg-3Ye-1La_2_O_3_ composites (UTS = 287.2 ± 4.4 MPa) was 55.1 MPa higher than that of Mg_3_Y alloy (UTS = 232.1 ± 2.6 MPa) by SEM-BSE, TEM and characterization experiments. Figure 1e,f shows that the strength of La and Ce MMCs is higher than that of Mg_3_Y alloys containing some other rare earth elements. Some Y will inevitably be consumed by La_2_O_3_, resulting in the formation of less fine Mg_24_Y_5_ phases and an increase in strength. The Coefficient of Thermal Expansion (CTE) of Mg and Mg_17_La_2_ are different. Due to the different cooling rate, more dislocations appear during the cooling process, so the dislocations can induce more uniform deformation, which results in a higher strength of Mg-3Ye-1La_2_O_3_ rich in Mg_17_La_2_. In addition, these dislocations make the plastic deformation more dispersed, thereby enhancing the matrix. The preparation of high purity rare earth elements required for WE alloys manufactured by conventional methods is very expensive. Therefore, some cheap and abundant rare earth oxides can be used as a candidate to replace the main elements in the corresponding alloys. A low-cost, high performance WE matrix composite was prepared by in situ synthesis. At present, the influence mechanism of rare earth on magnesium matrix composites is still not clear. Under extreme conditions such as additive manufacturing, the preparation of WE MMCs will have some special properties and discoveries [30]. This method can realize the industrialization of expensive rare earth magnesium alloys, and has broad prospects.

WE series alloys have high temperature wear and corrosion resistance. However, the high activity of magnesium makes the alloy have a strong ability to absorb O_2_, so some oxide impurities inevitably exist [31]. WE series alloys have additional rare earth losses and an increased cost. The commonly used method is the addition of grain refiners [31]. The in situ synthesis of MMCs can avoid the problem of rare earth losses to a certain extent, and can obtain high-performance magnesium-based MMCs. So far, reports indicate that the focus of WE alloy development is mainly Mg-Gd and Mg-Y. The development of rare earth resources is unbalanced; China is rich in La and Ce reserves, but they cannot be utilized. The reinforcement (La_2_O_3_) has the potential to improve the strength of the Mg-3Ye-1La_2_O_3_ alloy. In addition to precipitation hardening and grain refinement, the combination of Mg and RE_m_O_n_ is also a potential method to improve the performance. The in situ reaction of Mg and RE_m_O_n_ produces Mg_m_RE_n_. The formation of different interfaces and dislocations can be affected due to some factors including different cooling rates, thermal conductivity, diffusion rates, and so on; different interfaces and dislocations will be generated, which have various effects on the comprehensive properties of the composites. The presence of MMCs plays a role in controlling the comprehensive properties of WE matrix and reducing the cost.

### 2.3. Research and Development of Mg-Li Matrix Composites

Mg-Li alloy acted as an ultra-light alloy series with low density has been applied in military, aerospace, electronics, medicine and other fields. However, the low modulus of the traditional Mg-Li alloy is an inevitable disadvantage as a light alloy. Its mechanical properties can be significantly improved by introducing high modulus reinforcement and coating. Lithium is the most active metal particle, and the reaction between the reinforcement and lithium has not yet been clarified. Sun et al. [32] prepared TiB_2_/LA103 (Mg-10Li-3Al alloy) composites containing Mg-TiB_2_/LA103 (Al-3.15%) and Al-TiB_2_/LA103 (Al-3.26%) by mechanical stirring casting method modified by ball milling pretreatment. Aggregates were found in the mechanical ball milling process of Mg-TiB_2_ composite, which was due to the partial oxidation phenomenon, which led to the formation of refractory MgO in the sub-core–shell structure and hindered the diffusion of particles. In Al-TiB_2_/LA103, TiB_2_ particles are uniformly distributed. In Mg-TiB_2_/LA103, TiB_2_ aggregates along the grain boundary edge, so Mg-TiB_2_/LA103 has a strong brittle fracture tendency at the grain boundary. The agglomeration of reinforced particles is more likely to occur in the matrix. The mechanical properties can be further improved by changing the element of the matrix and effectively controlling the interface microstructure Li et al. [33] prepared Mg-5Li-3Al-2Zn (LAZ532) alloy matrix composites reinforced with La_2_O_3_ particles. The results indicated that UTS and ultimate compressive strength (UCS) reached 340.1 MPa and 415.5 MPa, respectively. The improvement in the comprehensive properties of the Mg-Li matrix composites should be attributed to the in situ formation of the Al_3_La phase.

Zeng et al. [34] fabricated an ultra-light Mg-10Li-3Al-3Zn (LA103Z) alloy by the integration of multi-directional forging (MDF), die forging and annealing treatment. The tensile yield strength (TYS) and the Ultimate Tensile Strength (UTS) of the alloys are 211 MPa and 243 MPa, respectively. Zhong et al. [35] added Y and Ce to the as-rolled Mg-8Li-1Al alloy, and its tensile strength reached 279 MPa. In contrast, the strength of the composite with light rare earth La is higher than that of the ordinary alloy material with heavy rare earth elements. Mg-Li composites have greater advantages in improving their strength and specific modulus. The evenly distributed reinforcing particles at the interface increase the density of dislocations and improve the properties of Mg-Li alloys. MMCs have a high potential for overcoming the shortcomings of the Mg-Li matrix itself, such as a low modulus, a mismatch between low density and high strength, high temperature strength, and stability. At present, there are few studies on Mg-Li matrix composites, and their related mechanism remains unclear.

### 2.4. Mg-Zr Matrix Composites

Mg-Zr alloy is regarded as a common wrought magnesium alloy, which was reported to possess a good plasticity and corrosion resistance. The application of an alloying element Zr as a grain refiner has an obvious grain refinement effect. At the same time, the Zr element can promote the anode reaction more easily, so the addition amount of the Zr element in the ZK alloy matrix needs to be strictly controlled. In the current research of magnesium alloys, the Zr element is commonly used in high temperatures and corrosion-resistant Mg-RE alloys for preventing the loss of Re and refining the grains, thus improving the strength, thermal conductivity and ductility of the alloy [36,37]. At present, the Mg-Zr alloy has the potential to be explored as an efficient reinforcing phase. Hussein et al. [38] prepared Mg and Mg-1Zr MMCs reinforced with sustainable and low-cost Eggshell (ES) using a powder metallurgy process. The corrosion rate of Mg-1Zr-2.5ES was 1.045, indicating its corrosion resistance was better than that of Mg-1Zr and Mg-2.5ES. It is verified that the combined effect of Zr and ES can more effectively strengthen the corrosion resistance of Mg nanocomposites. Shahin et al. [39] prepared Mg-0.5Zr-0.1GNPs Mg-Zr matrix nanocomposites by high-energy ball milling. The electrochemical corrosion potential of pure Mg, Mg-0.5Zr and MMCs were 1624.5 ± 21.0, 1602.4 ± 26.0 and 683.1 ± 24.6, respectively. This way of MMCs enhanced the corrosion resistance of Mg-Zr matrix. Otherwise, ES and MMCs also improved the YS and UTS of Mg-Zr matrix.

Magnesium alloy has strong biocompatibility, but its low corrosion resistance limits its application in biomedical fields. To improve the corrosion resistance of magnesium alloys or MMCs, rare earth elements are often introduced into the alloys and composites. The coordination of the traditional Mg-Zr alloy with the Re element has been extensively explored, and the research on the medical application of Mg-Zr MMCs is also on the rise. The corrosion resistance of Mg-Zr composites is comparable to that of WE composite, and its biocompatibility is better. Therefore, the Mg-Zr matrix has a strong research potential in the medical field.

### 2.5. Comparison and Summary of Various Matrixes in Magnesium Matrix Composites

As mentioned above, several MMCs substrates have their own unique features. Table 1 shows the comprehensive properties of the matrix in several common magnesium matrix composites. The AZ matrix, as a commonly used alloy material, has sufficient raw materials, low price and weak mechanical properties, but its corrosion resistance is weak. Through the production of MMCs to improve its corrosion resistance and mechanical properties, it is possible to achieve large-scale industrial production. The combination of the technical advantages of additive manufacturing and the high-performance characteristics of MMCs has a significant potential to promote the design and application of high-performance MMCs [2]. AZ matrices can be used as raw materials for additive manufacturing because of their good plasticity. The comprehensive performance of a WE-based matrix with good strength, thermal conductivity, and corrosion resistance is better than that of an AZ-based matrix. A WE-based matrix is used in aerospace and defense industries because of its excellent performance, but its cost is high. The production of MMCs is expected to reduce the cost of the WE-based matrix. Mg-Li alloys have low strength and good shock absorption performance, though their density is very small. The cooperative production of MMCs and rare earth elements can achieve the synergistic improvement in lightweight and high strength. The addition of a small amount the Zr element to the traditional Mg-Zr matrix has a strong grain refinement effect. However, the MMC-reinforced Mg-Zr matrix shows strong corrosion resistance, and has great potential in the medical field. Because the Mg-Zr matrix has a density similar to human bone, and can be absorbed by the human body, it is an excellent material for the 3D printing of human bones [40,41]. In order to meet the performance requirements of composite materials at different environments, composite materials need to have different matrix types. The rise of big data provides an effective toolbox for the screening of composite matrices, and the design of various matrices takes less time. MMCs increase the upper limit of the matrix. However, as the basis of MMCs, the matrix still has room for development.

## 3. Reinforcements of Magnesium Matrix Composites

The further application of magnesium alloys faces challenges due to its relatively low strength, poor corrosion resistance and insufficient high-temperature mechanical properties. Development of the metal matrix composite used magnesium as the base metal has the potential to overcome these shortcomings [4]. Magnesium matrix composites reinforced with metal-, ceramic- and carbon-based materials have bright prospects [5]. Strength and stiffness are improved by transferring the stress between the matrix and the reinforcement, and the elastic modulus and wear resistance are also improved [4]. Through a full combination of the reinforcement and the matrix, the strength of the reinforcement and the ductility of the matrix can be exerted [42]. This section outlines the reinforcement of MMCs from the aspects of the selection of reinforcement, the physical properties and comparative analysis of the main ceramic strengthening phases, and the particle size of the reinforcement.

### 3.1. Selection of Reinforcements

The selection of reinforcements should be based on good wettability, appropriate difference in CTE and excellent physicochemical compatibility with the matrix. Excellent mechanical properties depend on the beneficial interfacial reaction between the reinforcement grain and the surface of matrix. The low corrosion and economy should also be considered.

Hybrid reinforcement is a new tactic to improve the properties of a matrix material by mixing two or more reinforcement phases into the matrix material [4]. However, there are few studies on magnesium hybrid composites. This sub-section will discuss metal, ceramic and carbon material reinforcement.

#### 3.1.1. Metallic-Reinforced Mg-Based Composites

Metal reinforcements cooperate with the generated secondary phase, which improves the toughness of the composite material due to their good wettability and inherent toughness. Therefore, it has the strengthening effect of both the particles and the second phase [5,43]. Recently, various metal-reinforced MMCs have been developed, such as Ti/Mg [44], Sb/Mg [45], NbB_2_/Mg [46], TC_4_/Mg [47]. These metal reinforcements have higher melting points, and can act as a nucleation center during solidification and acquire minute equiaxed grains [5].

**Figure 2:**
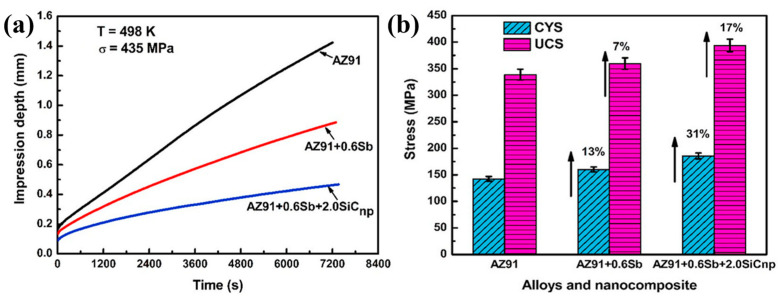
Creep and compression properties. (**a**) Creep indentation depth curves of each material at a certain temperature and pressure; (**b**) test results of compressive properties of alloys and composites [45].

Wang et al. [44] obtained uniform distribution of GNP and Ti mixed powder on the surface of AZ91 sheet by electrophoretic deposition and spraying. The uniaxial tensile test results confirm that the composite has excellent and comprehensive mechanical properties. The YS, UTS and elongation reach 160 MPa, 243 MPa and 20.8%, respectively. The high ductility of composites due to high activities of non-substrate slip and lamellar structure, and the addition of Ti particles are also beneficial to improve the plasticity. Ganguly et al. [45] added 0.6 wt% Sb and 2.0 wt% SiC (1.2 vol%) nanoparticles to AZ91 alloy by squeeze casting. The results (Figure 2) demonstrate that the addition of nanoparticles reduces the average grain size and the percentage by volume of β-Mg_17_Al_12_ phase, and thus enhances the compressive strength and creep resistance. Luo et al. [47] prepared Ti-6Al-4V (TC_4_) particle reinforced composites by powder metallurgy and hot extrusion. When the content of TC_4_ particles was increased to 5 wt%, the composite reached maximum YS (211 MPa), UTS (303 MPa) and elongation (18.7%). Compared with the AZ91 alloy, the elongation and UTS of the composites increased by 18.7% and 303 MPa, respectively. At the same time, it can be found that TC_4_ particles can alleviate stress concentration and improve work hardening ability.

Table 2 compares the comprehensive properties of some common metal-reinforced MMCs. Titanium and its alloys as reinforcements can effectively increase the strength of magnesium-based materials. This way, good compatibility can be achieved because of their similar crystal structure. Therefore, the high-melting-point metal Ti is considered to be a potential reinforcing material [47,48,49]. Moreover, Ni metal particles with a high melting point [50] or a Ti-Ni shape memory alloy [49] used as reinforcement have a better enhancement effect. High-entropy alloys (HEA), as an emerging alloy, have also attracted widespread attention. For example, adding iron greatly increases the strength of the materials [51,52].

#### 3.1.2. Ceramic-Reinforced Mg-Based Composites

Ceramic reinforcements are of the highest interest as reinforcement materials because of their relatively high modulus, high hardness, low CTE, good high temperature performance, excellent thermal stability and high chemical stability. Ceramic reinforcements can refine the magnesium matrix grains, produce high-density dislocation, and significantly improve strength. If the ceramic reinforcement has a regular and uniform shape, the generated residual stress is very small. The main ceramic reinforced materials include carbide (SiC, ZrC, WC and TiC), metal oxides (La_2_O_3_, Y_2_O_3_ and Bi_2_O_3_), nitrides (Si_3_N_4_, TiN, ZrN and AlN), and borides (VB_2_, NbB_2_ and TiB_2_) [5]. Table 3 gives a summary of the comprehensive properties of some representative ceramic reinforced materials. By comparison, each ceramic particle with a high melting point and an on-line expansion coefficient without a significant difference has the potential to enhance high temperature mechanical properties. In addition, most of them have a hexagonal structure, which can form a good match with the magnesium matrix to improve the compatibility. Especially, AlN exhibits an excellent thermal conductivity, which is about five times that of other materials.

However, the increase in tensile strength comes at the cost of sacrificing elongation due to the brittleness of the ceramic itself. This phenomenon is attributed to the difference in the crystal structure and the low wettability between the reinforcement and the matrix. Nano-sized ceramic reinforcements significantly improve the strength and plasticity of the composites. However, compared with metal, nano-ceramic reinforcements are prone to agglomeration and it is difficult to achieve uniform dispersion. In addition, other shortcomings, such as poor interfacial adhesion caused by the reaction between the reinforcement and the matrix, result in decreased performance and interface stability [5].

#### 3.1.3. Carbon-Reinforced Mg-Based Composites

One-dimensional carbon nanotubes and two-dimensional graphene nanosheets with attractive characteristics in mechanics, electricity and heat are attractive reinforcements for fabricating metal–matrix composites. Compared with ceramic and metal particle reinforced materials, nano-carbon-reinforced MMCs have lower density, higher specific strength and higher stiffness. In addition, CNT and GNS also exhibit high Young’s modulus, better hardness, compression and bending resistance, and excellent thermal stability. The high modulus of nano-carbon reinforced materials can also achieve second phase strengthening.

Saikrishna et al. [3] prepared multi-walled carbon nanotubes (MWCNT) reinforced MMCs by friction stir processing (FSP) and measured the microhardness of the materials. It was found that the composites microhardness was greater than that of pure magnesium samples. Electrochemical studies have shown that adding CNT will improve the corrosion performance of the composites. Kandemir et al. [54] prepared recycled short carbon fiber composites using high shear dispersion technology. The Vickers hardness (HV) test shows that the hardness of the alloy increases by 6.5% and 13% for the fiber with a length 100 μm and 500 μm, respectively. The results show that the presence of carbon fiber significantly enhances the compressive properties of the composites, while the minimum creep rate has not increased. Meng et al. [55] prepared graphene nanoplatelets (GNPs) reinforced magnesium-laminated MMCs by hot pressing and rolling. The results show that the composite with 0.25 vol% GNP exhibits a tensile strength of 160 MPa, which is lower than that of composite with 0.75 vol% GNP (179 MPa) and higher than that of pure magnesium (136 MPa). At the same time, GNPs slow down crack propagation and maintain the ductility of the composite.

**Figure 3:**
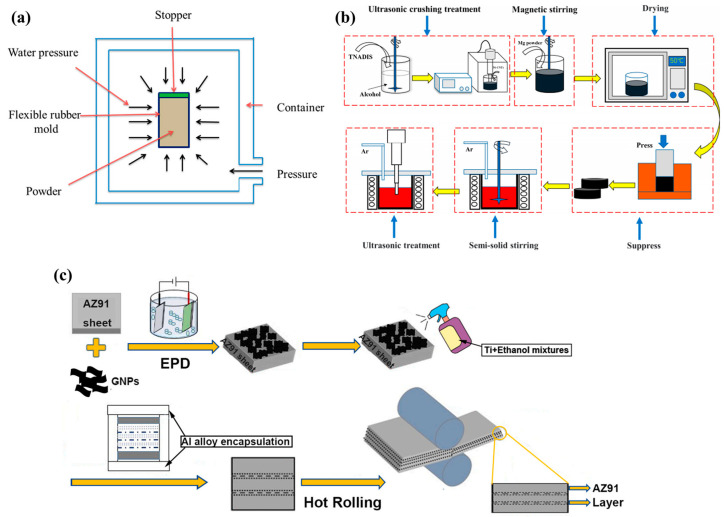
Methods of homogeneous dispersion of reinforcements. (**a**) Powder metallurgy [56]; (**b**) ultrasonic combined with semi-solid stirring [57]; (**c**) laminated composite process [44].

Similar to ceramic reinforcements, the agglomeration and uneven dispersion of carbon reinforced materials can also lead to a decline in their performance. Figure 3 gives several methods of homogeneous dispersion of reinforcements. Friction stir processing (FSP) is a promising method for solid-state processing that can achieve homogeneous distribution of CNT on surfaces without melting the matrix material [3]. The powder metallurgy (PM) process requires fewer complex steps to achieve the dispersion of a larger number of nanoparticles, while being more economical and saving more time and materials [56]. Ultrasonic treatment assisted with semi-solid stirring can break agglomerations by ultrasonic waves [57]. The laminated structure can provide lower cost and better performance, which is beneficial to facilitate uniform reinforcement distribution in large MMCs [44]. If continuous and uniform distribution can be achieved, the mechanical properties of CNT or graphene reinforced composites will exceed those of carbon fibers. In addition, the weak interfacial adhesion between CNT and matrix reduces the performance of nano-carbon reinforced magnesium matrix composites. The non-wettability leads to the uneven distribution of reinforcements. Coating the surface of nano-carbon is proven to be one of the best technologies for improving the interfacial bonding and wettability between the reinforcement and the matrix.

No matter what kind of reinforcement material is used, it is very important to realize the uniform dispersion of particles, and it is also an urgent problem to be solved. Solving the interface diffusion issues, exploring the interface mechanisms, and finding a suitable interface optimization method should be the direction of the next stage of experimental exploration. In addition, a reduction in the reinforcement grain size by the replacement of micron-sized particles by nano-sized particles is a common development trend of various reinforcements. Further optimization of the manufacturing process to achieve lower cost and higher efficiency still needs further research. Metal reinforcements may increase the density of magnesium matrix composites, so more composites based on magnesium alloys need to be developed. As for ceramic reinforcements, the synergistic effect of various ceramic reinforcements is worth further study.

### 3.2. The Physical Properties and Comparative Analysis of the Main Ceramic Reinforced Phases

#### 3.2.1. Alumina (Al_2_O_3_)

Alumina is one of the most employed ceramic materials, showing an excellent combination of properties that includes high hardness, good friction and wear properties, and excellent chemical compatibility with the matrix [4]. Azizieh et al. [58] prepared AZ31/Al_2_O_3_ magnesium matrix nanocomposites by friction stir processing. They found that the hardness of composite (70~90 HV) was greatly improved compared with that of the matrix (50 HV) because of the grain refinement and the presence of hard reinforced particles Al_2_O_3_. It was found that the addition of nano-Al_2_O_3_ reduced the plastic deformation while providing a load-bearing capacity and altered the wear mechanism of the material so that the rate of wear of the nanocomposites was lower than that of the matrix metal, which may be attributed to the fact that nanocomposites with a higher hardness limited the flow of the material. Thirugnanasambandham et al. [59] fabricated Al_2_O_3_ nanoparticle-reinforced magnesium matrix composites by a stir casting technique. The wear test indicates that the presence of Al_2_O_3_ improves the wear resistance of magnesium alloy by 28%, and increases the friction coefficient from 0.095 to 0.55. The improvement in wear resistance is related to finer grains and excellent thermostability. The influence of sliding speed on the wear behavior in the wear process is also worthy of attention because the oxidation effect increases with increasing the speed. At the same time, Al_2_O_3_ can also improve the corrosion resistance of the matrix due to its chemical inertness. If the reinforcement further solves some key problems in biological activity, which is beneficial to realize the potential of magnesium matrix composites in medical bone implants.

#### 3.2.2. Silicon Carbide (SiC)

SiC is a wide band gap semiconductor, and because it has stable chemical properties, high thermal conductivity, small thermal expansion coefficient, good wear resistance and relatively low cost, SiC nanoparticles incorporated in magnesium can significantly enhance the tensile properties of magnesium by significantly refining the grain size. Unfortunately, nano-SiC often shows aggregates in Mg matrices. Compared with nanoparticles, submicron particles are easier to disperse and increase in the number of particles relatively, thereby obtaining better tensile properties while simultaneously sacrificing their ductility.

**Figure 4:**
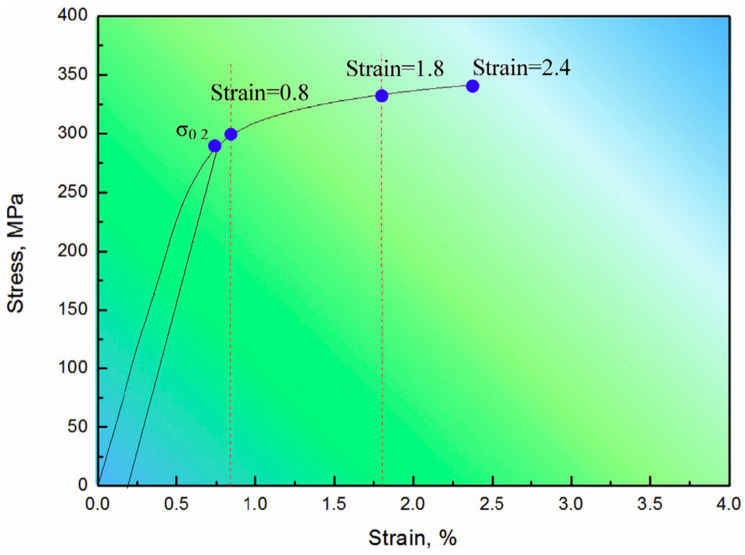
The strain–stress curve of double-size SiCp/Mg composites [60].

Shen et al. [60] prepared 1 vol% nano-SiC particles and 4 vol% sub-micron SiC particles reinforced double-size SiC_p_/Mg composites by a combination of stir casting and hot extrusion. It was found that the synergistic effect of the two SiC particles can increase the dislocation density, inhibit the movement of the dislocation and transfer the load more effectively, which is beneficial to significantly improve the tensile strength (TS) of the composite (Figure 4). Guo et al. [61] found that the addition of SiC nanoparticles had a significant effect on the microstructure of Mg-25Zn-7Al (wt%) alloy during solidification, in which the grain size was significantly reduced, the growth morphology of dendritic grains was changed to a hyperbranched morphology with more split tips, which was more robust.

Specially, the analysis of the contribution of various strengthening mechanisms (load transfer, Hall–Petch, dislocation density increase, Orowan, thermal expansion coefficient mechanism) of SiC reinforcement to the strength of magnesium matrix composites is also the focus of research. At the same time, the synergistic strengthening effect of SiC with different sizes deserves further attention.

#### 3.2.3. Titanium Diboride (TiB_2_)

TiB_2_ ceramic particles have received extensive attention due to its excellent properties such as high melting point, high hardness, high modulus of elasticity, high thermal stability, low density, good wear resistance and chemical stability. Meher et al. [62,63] produced TiB_2_-RZ5 metal matrix composites by SHS route. The results show that the hardness and strength of the composites increase with increasing particle concentration. The ultimate tensile strength of the composites with 8 wt% TiB_2_ increases by 30.47% and the hardness increases from 64.2 VH to 85.0 VH. At the same time, the increment of TiB_2_ concentration in the matrix gradually improves the resistance of the material to plastic deformation and the wear resistance, simultaneously the wear loss and friction coefficient of the material are also reduced. Xiao et al. [64,65] prepared 2.5 wt% nano-TiB_2_/AZ91 composites by adding an intermediate alloy to molten magnesium. Through the Brinell hardness test and tensile test, it was found that the hardness of the composite was remarkably enhanced compared with the matrix alloy. Especially, the yield strength, the UTS and the elongation at break were increased by 49.4%, 25.5%, and 51.1%, respectively. Simultaneously enhancing strength and ductility may be largely attributed to grain refining and the uniform distribution of TiB_2_ reinforced particles.

TiB_2_ is considered to be one of the most promising reinforcement materials because it has the same crystal structure with magnesium and a high lattice matching degree. The added TiB_2_ can be used as a grain refiner, which effectively enhances the mechanical properties of the material. However, excessive TiB_2_ contents will lead to severe brittleness, which is not beneficial to practical application. In situ synthesis of TiB_2_ reinforced magnesium matrix composites is a promising research hotspot. This method can provide a clearer interface between the reinforcement and the particles, better thermodynamic stability and finer reinforcement size.

#### 3.2.4. Aluminum Nitride (AlN)

AlN ceramic particles exhibit a series of advantages such as high hardness, high melting point, high strength, good thermal conductivity, low density and low expansion coefficient. At the same time, AlN has the same crystal structure and similar lattice parameters with Mg, and has a high lattice matching. Sun et al. [66] measured the microhardness of the material by a Vickers hardness tester. It was found that the peak-aged AlN/AZ91 composite obtained a 14% elongation at break and a tensile strength of 275 MPa. Compared to the base alloy, the strength and elongation were increased by 44% and five times, respectively. Yang et al. [67] prepared in situ AlN reinforced magnesium matrix composites by nitridation reaction. As shown in Table 4, the compressive yield strength, ultimate compressive strength and compressive elongation of AIN reinforced AZ91 composites with volume fraction of 1.5 vol% are 110 MPa, 371 MPa and 22.1%, respectively, achieving simultaneous improvement in compressive strength and plasticity.

Adding AlN not only improves the mechanical properties of the metal matrix, such as strength, hardness and plasticity, but also plays a unique role as a functional material in the electronic packaging industry and other fields. Similar to TiB_2_, in situ synthesis also provides a feasible high comprehensive performance development path for AlN-reinforced magnesium matrix composites, which can achieve a synchronous increase in the strength and ductility of the composites.

#### 3.2.5. Tungsten Carbide (WC)

Tungsten carbide (WC) nanoparticles are a widely used engineering material due to their excellent properties such as high elastic modulus, high oxidation resistance at high temperature, high melting point and high hardness, good thermal conductivity, good shock absorption ability and good anti-corrosion ability. The positive contribution of nano-WC to the mechanical and tribological properties of Al/Mg composites has been reported.

Banerjee et al. [68] prepared AZ31 magnesium alloy matrix nanocomposites with WC by ultrasonic vibration assisted stirring casting method, and they found the microhardness of the composite increased with increasing nano-WC content. The electrochemical impedance spectroscopy and potentiodynamic polarization tests revealed that the Mg matrix nanocomposites with 0.5 wt% WC had the strongest corrosion resistance, indicating that the addition of WC may improve the corrosion resistance and the surface roughness. Banerjee et al. [69] prepared WC nanocomposites with varying weight percentages by ultrasonic vibration-assisted stirring casting, and they found the addition of WC nanoparticles increased the hardness of the composites. The analysis of the friction and wear behavior at room temperature under dry sliding conditions indicated that the incorporation of WC nanoparticles enhanced the wear properties of the composites.

In recent years, the parameter optimization algorithm is commonly used in WC/Mg matrix composite system. For example, Banerjee et al. [70] used Taguchi method to study the wear properties of nano-WC reinforced magnesium matrix composites. Karuppusamy et al. [71] studied the impact of treated WC on the wear properties of magnesium alloys by multi-level factor design. Kumar et al. [72] proposed a league championship optimization (LCO) technique to select the composition parameters of the Mg-WC metal matrix.

#### 3.2.6. Bio-Ceramic Reinforcements

Hydroxyapatite (HAP) is one of the most sought after biomaterials due to its likeness with natural bone components, which is beneficial to osteogenesis. Cui et al. [73] successfully prepared Mg-Zn/HA composites by spark plasma sintering (SPS). The compressive yield strength improves with the increment of HA content. The compressive yield strength and flexural strength of the composite with 10 wt% nano-HA were increased by 43% and 14%, respectively, compared with that of the matrix alloy. The anti-degradation ability was increased by 49%. In vitro experiments showed that the composite had a good cell compatibility.

**Figure 5:**
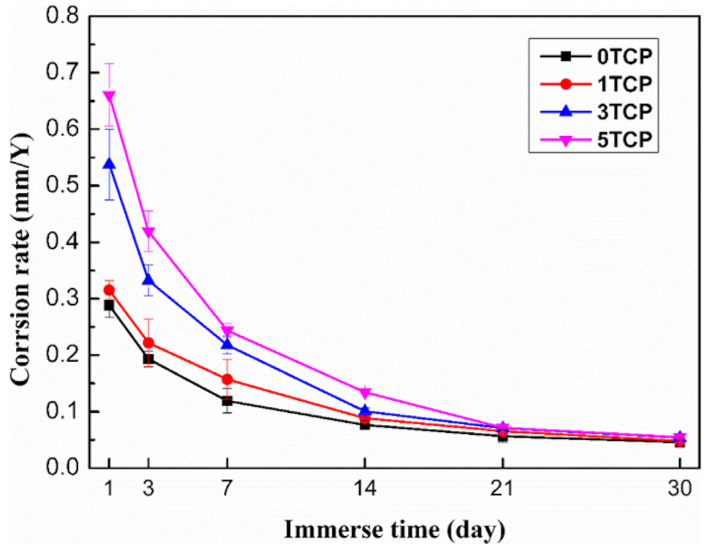
Corrosion rate of composites with different TCP content [74].

β-tricalcium phosphate (β-TCP) is also one of the most widely studied biomedical reinforcing materials due to its higher dissolution rate. Its composition is similar to human bone, so it has good bone conductivity, biocompatibility, biological activity, biodegradability and biological absorption properties. At the same time, as a bioactive ceramic, it significantly improves the mechanical properties of the composite. Pan et al. [74] prepared β-TCP reinforced magnesium–aluminum alloy matrix composites by mechanical stirring and ultrasonic assisted casting. The results indicated that the YS, UTS and elongation of Zn-Mg/β-TCP composites reached 250.8 MPa, 330.5 MPa and 11.7%, respectively. However, the increases in β-TCP concentration sacrifices part of the corrosion resistance of the material (Figure 5). Comprehensive cytotoxicity tests and animal experiments show that the composite material has better biocompatibility and higher safety than that of the matrix alloy.

Bio-ceramics HAP and TCP are more concerned due to their corrosion resistance and biocompatibility. Biodegradable metal demonstrates good mechanical and biological properties in bone implants. As a biodegradable implant, the exploitation of bio-ceramic-reinforced magnesium matrix composites has become an essential research field in medicine. The addition of ceramics can avoid the toxicity and performance degradation caused by the addition of elements such as Al, Re, Ca and Fe. It cannot continue to play a role before the tissue repair is completed if the corrosion resistance is too poor, which will lose its significance. It is very important for them to maintain mechanical integrity for a certain period of time because they need to provide complete support throughout the healing process.

#### 3.2.7. Other Reinforcements

In addition to the five above-mentioned ceramic particles, BN, B_4_C, Si_3_N_4_, ZrSiO_4_, La_2_O_3_, Y_2_O_3_, Bi_2_O_3_, etc. can also be used to enhance the magnesium matrix.

Mohammadi et al. [75] fabricated magnesium matrix composites reinforced with B_4_C particles with different contents by casting. Their results indicate that the addition of B_4_C particles slightly increases the UCS, yet leads to a decrease in UTS and elongation. Xiao et al. [76] prepared LAZ532 alloy matrix composites reinforced by La_2_O_3_ particles via melt casting. It was found that the composites exhibited excellent quasi-superplasticity, and the elongation at break achieved 213% at 573 K and a strain rate of 8.3 × 10^−5^ s^−1^. Ghasali et al. [77] observed that porous Mg-Si_3_N_4_ nanocomposites prepared by microwave sintering had a higher polarization resistance than that of pure magnesium, which increased the corrosion resistance of the matrix.

### 3.3. Effect of Ceramic Particle Size on Microstructure and Properties of Magnesium Matrix Composites

The dimension and shape of the ceramic reinforcements (particles, whiskers or fibers [4]) have a great effect on the performance of MMCs [5]. The weight or volume fraction of the particle inclusions is usually very high at a micrometer-scale size, which is intend to form clusters to affect the wettability, resulting in poor ductility and a reduction in the life of the composite [69]. At the same time, the crack preferentially appears around the micron particles compared to the nanoparticles when the applied stress exceeds the strength of the material, which is correlated to the lower bonding strength caused by the greater stress concentration between the micron-sized reinforcement and the matrix interface. In addition, submicron particles are easier to disperse in the magnesium matrix, which can increase the number of particles and improve the strength of the material, yet reduce the ductility [60]. With the development of nanoscience, micron particles are gradually replaced by nanoparticles [68]. Recent studies have shown that nanoparticle-reinforced composites have lighter weight, higher strength and better wear resistance [5]. By reducing the particle size, the grain size is significantly reduced [61] and the mechanical properties such as hardness, ductility, strength and tribological properties [64,69] are further considerably improved. Unfortunately, the nanoparticles cannot be uniformly distributed because of the aggregation in the matrix [68,69]. This is mainly due to its high surface energy, which also limits the number of nanoparticles and cannot achieve the continuous improvement in the comprehensive performance of the composite [60]. In addition, the potential health effects of nanoparticles on different organ systems are not yet fully understood [54].

## 4. Interface Design and Control between Reinforcement and Magnesium Matrix

MMCs are mainly composed of a magnesium matrix, a reinforcement, and the interface between them [78]. In MMCs, the addition of these reinforcements improves the performance of MMCs through load transfer, grain refinement, induced crack deflection and obstruction of dislocations [79]. Among them, load transfer is the main hardening mechanism of MMCs. The interface between the magnesium matrix and the reinforcements is a bridge that maintains the transfer of load from the matrix to the reinforcement. The interfacial bonding strength determines the final mechanical properties of MMCs to a large extent [78,80]. Therefore, it is a key issue to study the improvement of the comprehensive performance of MMCs from the perspective of interface regulation. This section mainly reviews the two critical factors affecting the interface bonding strength and the methods for improving the interface bonding.

### 4.1. Factors Affecting the Interface between Reinforcement and Magnesium Matrix

#### 4.1.1. Wettability

A better wettability means that the molten magnesium matrix can better encapsulate the solid reinforcement, thereby improving the comprehensive properties of the MMCs. For nanoparticle-reinforced magnesium-based alloys, the presence of nanoparticles further improves the comprehensive properties of MMCs, effectively balancing the contradiction between strength and plasticity [81]. However, it is usually difficult to achieve better wettability between the molten magnesium matrix and ceramic reinforcement due to the high surface tension of the molten matrix phase. In addition, the agglomerates of nanoparticles in the magnesium matrix cause coarse grains, and thus leads to a reduction in the properties of nanoparticle-reinforced magnesium matrix composites.

Wettability is commonly characterized by the contact angle (θ). The smaller contact angle (θ < 90°) indicates that the interface between the matrix and reinforcement has a better wettability; that is, the molten metal matrix can better infiltrate the surface of the reinforcement particles. For example, a high solid–liquid interfacial energy between magnesium alloy and various ceramic materials such as TiC, Si_3_N_4_, and SiC [82] results in a large contact angle between the magnesium matrix and ceramic nano-reinforcement, which limits the further enhancement of the higher volume fraction of the reinforced grains in the MMCs [83]. In addition, the passive oxidation of silicon nitride was used to reduce the contact angle between the liquid magnesium matrix and silicon nitride, which enhanced the interfacial wettability of the magnesium matrix composites and promoted the improvement in the comprehensive properties of the Mg-AZ91E alloy [83].

In other words, in order to fabricate the MMCs with excellent mechanical properties, the main issue to be faced is the poor wettability between the reinforcements and molten magnesium matrix. The new casting methodologies need to be explored in the future.

#### 4.1.2. Reactions between Reinforcement and Magnesium Matrix

As mentioned above, the load transfer is an important strengthening and toughening mechanism in nanoparticle-reinforced magnesium-based alloys. Therefore, a strong transition interface between the nano-reinforcement and the matrix is required to form a strong bond. In addition to the wettability between the nano-reinforcement and the matrix, a suitable interphase formed by the chemical reactions between the two components enhances the interfacial bonding [84]. This interfacial reaction is also the focus of our discussion.

As an active metal, magnesium is always inevitably accompanied by different degrees of interface reaction [78], dissolution, diffusion and segregation of elements in the process of preparing MMCs, forming the interfaces with different structures and bonding strength [85]. These interfaces are unstable, which will induce interface reaction under certain conditions.

**Figure 6:**
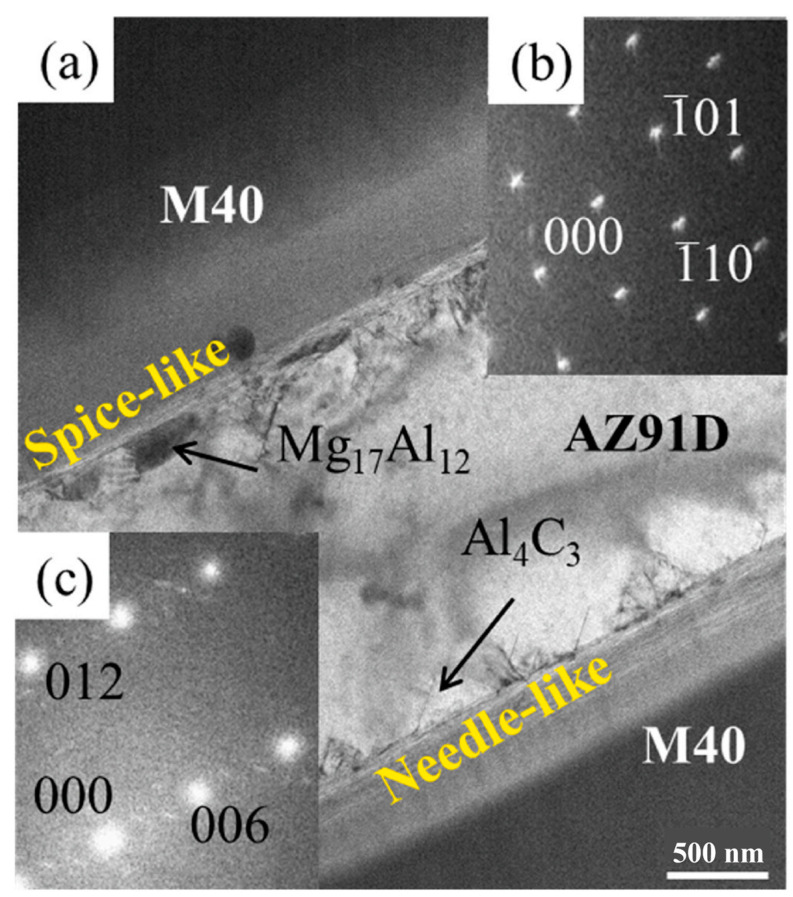
The TEM observation of C_f_/Mg composites. (**a**) Bright field transmission electron microscopy was used to observe the interface; (**b**) Mg_17_Al_12_ under the SAED mode; (**c**) Al_4_C_3_ under the SAED mode [86].

**Figure 7:**
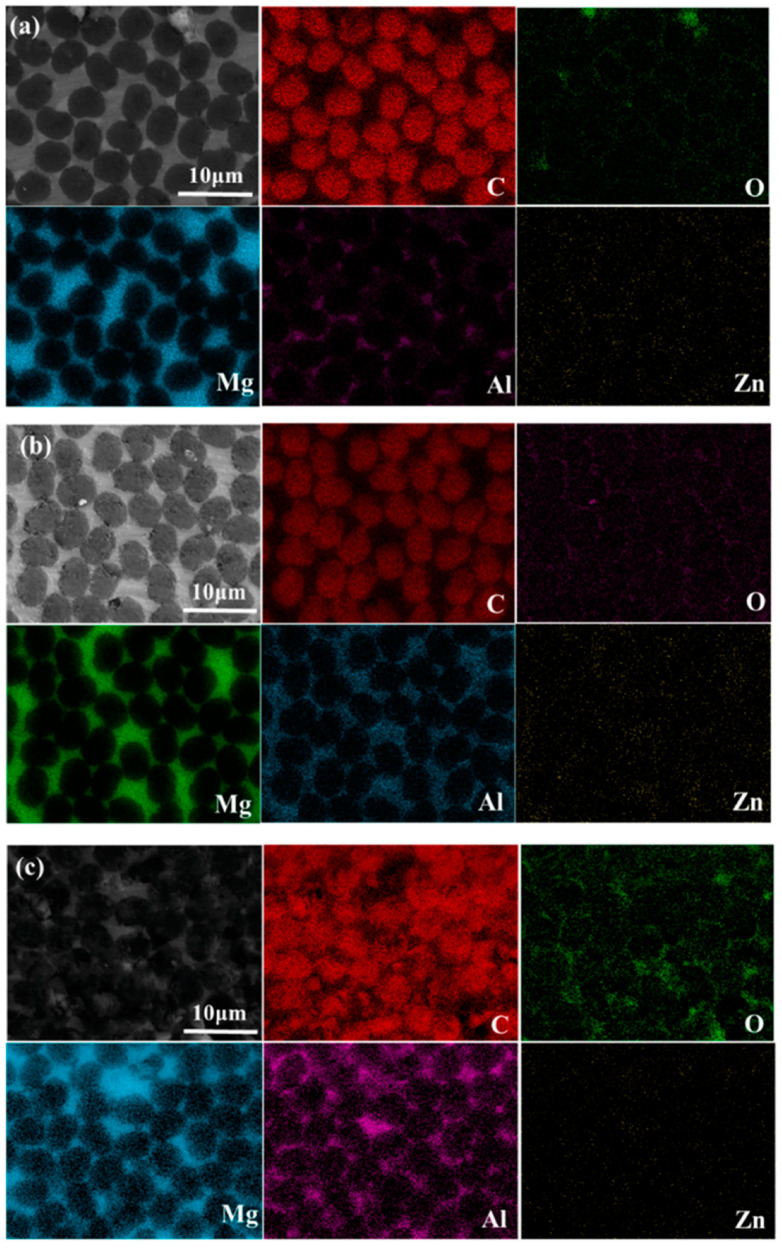
The element distributions of C_f_/Mg composites under different casting temperatures (CAT). (**a**) 750 °C; (**b**) 760 °C; (**c**) 780 °C [86].

**Figure 8:**
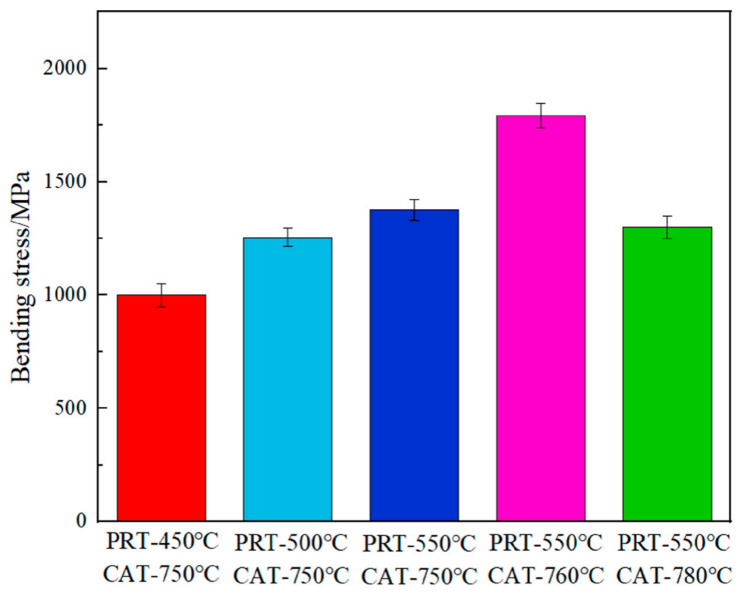
The bending strength of C_f_/Mg composites under different preparation temperatures (PRT) [86].

On the one hand, the appropriate weak interfacial reaction can improve the wettability between the reinforced particles and the molten metal. On the other hand, the brittle phase precipitations caused by chemical reaction greatly aggregate at the interface to form a brittle layer [78], which may reduce the bearing capacity of the material the comprehensive mechanical properties of the composite. Sun et al. [86] prepared M40/AZ91 composites by pressure infiltration and investigated the effects of preheating temperature (PRT) and casting temperature (CAT) after AZ91D melting on the interfacial reaction and comprehensive properties of C_f_/Mg composites. As shown in Figure 6, Al_4_C_3_ and Mg_17_Al_12_ were formed through the interfacial reaction. These two precipitates were beneficial to the effective load transfer between the matrix and the reinforcement, improving the bonding strength of the interface. As shown in Figure 7 and Figure 8, the intensity of interface reaction increases with the increase in PRT and CAT, and the serious separation of aluminum element is observed on the surface of the fiber, which reduces the bending strength and mechanical properties of the material. Therefore, only a moderate interfacial reaction can enhance the interfacial adhesion strength and further improve the comprehensive properties of MMCs.

In summary, improving the wettability and interfacial reaction are two important paths to regulate the comprehensive properties of nano-reinforced MMCs.

### 4.2. Methods to Improve the Interfacial Bonding Strength of Nano-Reinforced Magnesium Matrix Composites

According to the composition of MMCs and the factors affecting the interfacial bonding strength mentioned above, the methods for designing and controlling the interfacial structure and properties of composites have been explored from the perspectives of alloying treatment of metal matrices, surface treatment of the reinforcements, and adjusting the parameters of the process.

**Figure 9:**
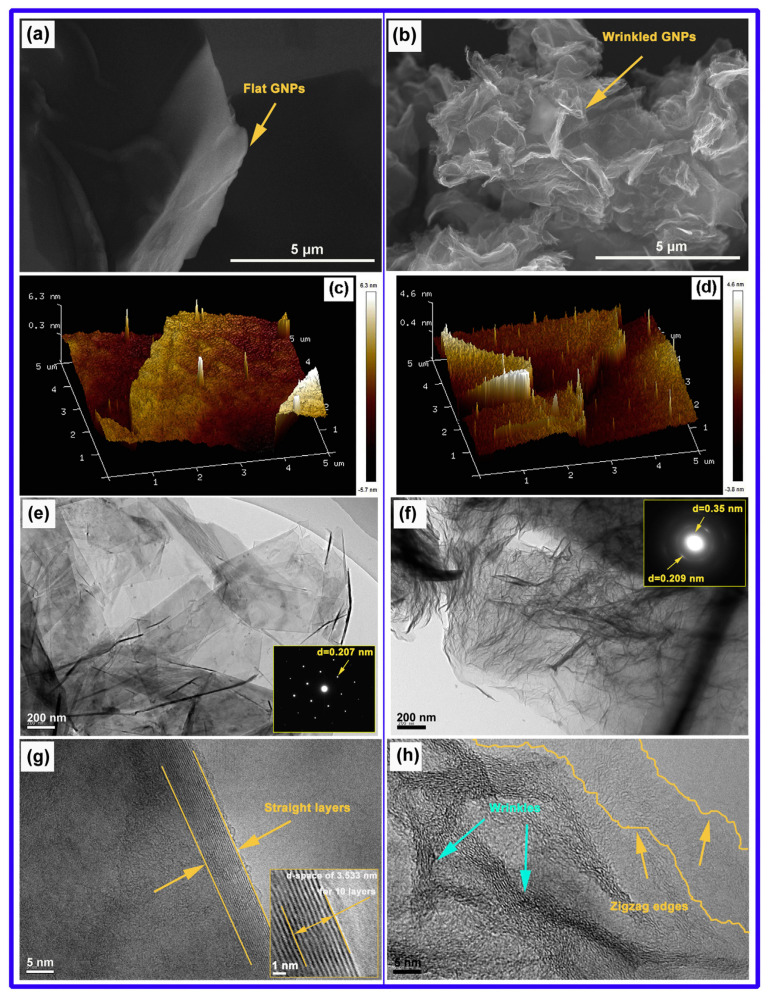
The images of GNP-1: SEM (**a**), AFM (**c**), TEM (**e**), HRTEM (**g**). The images of GNP-2: SEM (**b**), AFM (**d**), TEM (**f**), HRTEM (**h**) [87].

**Figure 10:**
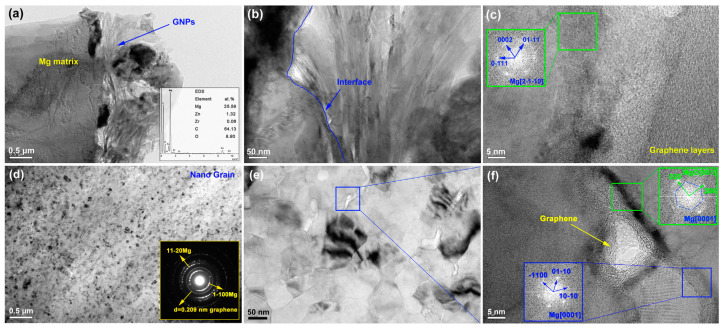
The interfacial conditions of GNP−1/ZK60 composite: (**a**–**c**), GNP−2/ZK60 composite: (**d**–**f**) [87].

**Figure 11:**
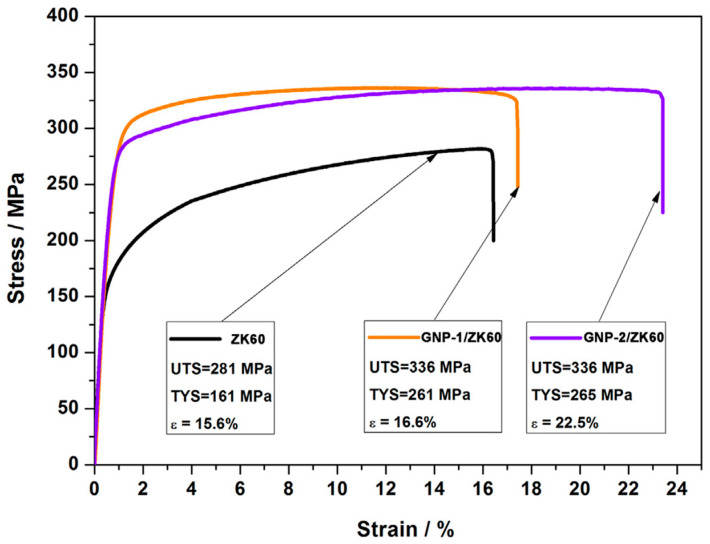
Tensile curves of different alloys and composites [87].

Du et al. [87] studied the defects in graphene nanoplatelets (GNPs) and their interface behavior of magnesium alloy through using two different structures of graphene nanosheets (GNP-1 has high integrity and GNP-2 has defects). As shown in Figure 9, compared to GNP-1 with a clean surface and high crystallinity shown on the left side of the picture, the defective GNPs exhibits carbon vacancies, broken carbon bonds, oxygen-containing functional groups and wrinkles with high specific surface area, and the jagged edges. As shown in Figure 10, the GNPs did not undergo a phase change while the magnesium atom reacted with oxygen in the chemical functional group to form MgO nanoparticles, forming a strong interfacial adhesion. In addition, the defects of graphene also trigger the diffusion nucleation of Mg nanocrystals, connecting hard GNPs with soft Mg matrix, thus effectively transmitting stress and strain. Du et al. [87] tested the tensile properties of ZK60, GNP-1/ZK60 and GNP-2/ZK60 composites at the strain rate of 0.003 s ^−1^. As shown in Figure 11, the tensile yield strength (TYS) of the two kinds of GNP reinforced composites is about 263 MPa, which is 63% higher than that of ZK60, whose tensile yield strength is about 161 MPa. At the same time, the ultimate tensile strength (UTS) is increased by nearly 20% compared with ZK60 without GNP enhancement. Compared to the ZK60 alloy, the elongation (%) of the GNP-2/ZK60 composite increases nearly 44%, which is much better than that of GNP-1 enhanced ZK60 alloy. This proves that the interface between the GNPs and the magnesium matrix makes the composite material a reliable entity, effectively ensuring the transmission of load and strain between the matrix and the reinforcement, and greatly improving the plasticity of the composite material.

**Figure 12:**
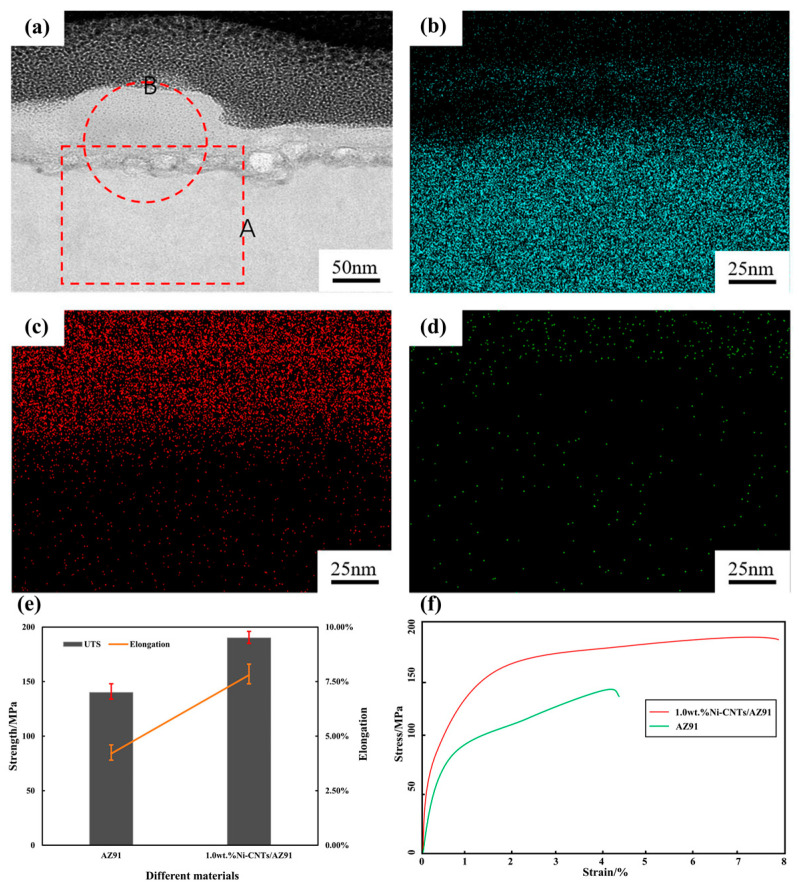
The distribution of CNTs, Mg and Mg_2_Ni. (**a**) TEM (Both A and B are regions on the interface); (**b**) EDS mapping of Mg; (**c**) EDS mapping of C; (**d**) EDS mapping of Ni (region A); (**e**) the tensile mechanical properties of AZ91 matrix material and 1.0 wt% Ni-CNTs/AZ91 MMCs at room temperature; (**f**) tensile stress–strain curve of AZ91 matrix material and 1.0 wt% Ni-CNTs/AZ91 MMCs at room temperature [57].

Liu et al. [57] prepared Ni-CNTs/AZ91 MMCs by ultrasonic treatment and semi-solid stirring method. Ni-CNTs were uniformly distributed in the Mg matrix, and good interface bonding was achieved. Meanwhile, the in situ formed Mg_2_Ni phase was observed at the interface between the matrix and carbon nanotubes (Figure 12a–d). They tested the tensile properties of AZ91 alloy and Ni-CNTs/AZ91 MMCs (Figure 12e,f). By comparison, the UTS of the Ni-CNTs/AZ91 MMCs increased from 140 MPa to 190 MPa, increased by 36%, and the elongation increased from 4.2% to 7.8%, increased by 86%. The remarkably enhanced mechanical properties were attributed to the strong interface bonding due to the in situ formed Mg_2_Ni phase.

Ding et al. [88] achieved uniform dispersion of CNTs in the matrix and obtained a strong interface bonding by surface treatment of carbon nanotubes (CNTs), such as electroless copper plating, ball milling, sintering and extrusion. The formation of a carbon nanotube interface phase due to the reaction between the carbon nanotube modified copper and the magnesium matrix, thereby improving the interface bonding. In addition, the uniform distribution of carbon nanotubes at grain boundaries hinders the discontinuous dynamic recrystallization process, which leads to the grain refinements. As a result, the strengthening and toughening efficiency of CNTs/Mg composite reach 325% and 264%, respectively, by adding 0.125 vol.% carbon nanotubes.

**Figure 13:**
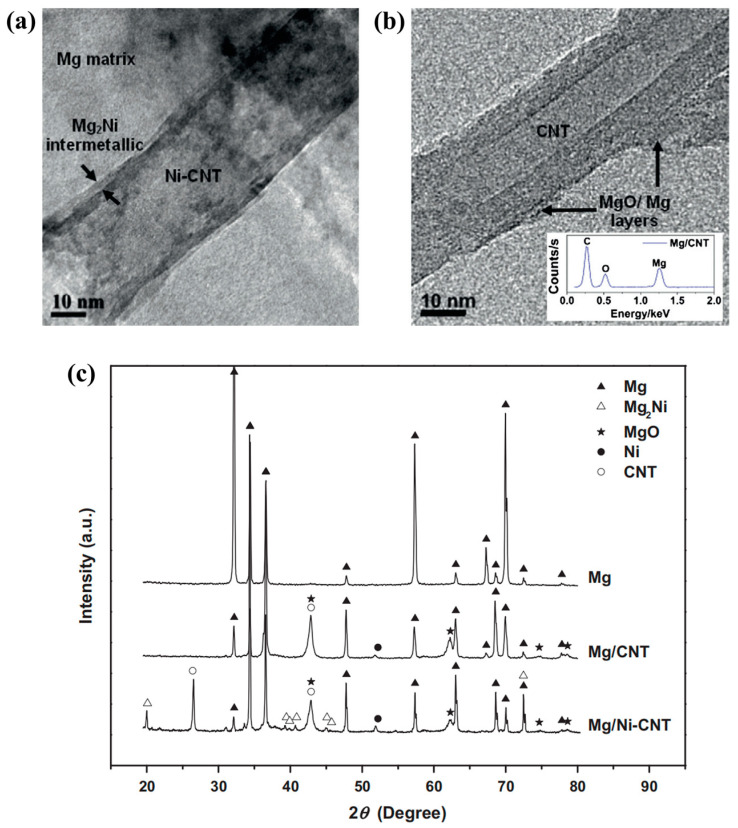
(**a**) TEM images of intermetallic compounds formed at the interface between Mg matrix and Ni-CNT; (**b**) TEM images of isolated CNT and EDX spectra of elements present on the surface of CNT; (**c**) XRD patterns of Mg, Mg/CNT and Mg/Ni-CNT [89].

Nai et al. [89] reported that the interfacial properties of magnesium composites were enhanced by using nano-thick metal Ni coating onto carbon nanotube reinforcements. As shown in Figure 13, an obvious reaction zone with the average thickness of 1.69 ± 0.5 nm can be observed, indicating that a continuous intermetallic phase at the interface the metal matrix composite is formed due to the reaction between the nickel coating reacts and the surrounding Mg matrix. In fact, the presence of a Ni-coated layer on the CNT surface leads to the formation of Mg_2_Ni intermetallic compounds at the interface, which improves the interfacial bonding strength for Mg/Ni-CNT composite, refines the particle size of the Ni-CNT reinforcement, and homogenizes its dispersion in the Mg matrix. Afterwards, they prepared Mg/CNT and Mg/Ni-CNT composites by powder metallurgy technology. The results indicate the microhardness, ultimate tensile strength and 0.2% yield strength of the Mg/Ni-CNT composites were enhanced by 41%, 39% and 64%, respectively, compared to that of the magnesium matrix.

**Figure 14:**
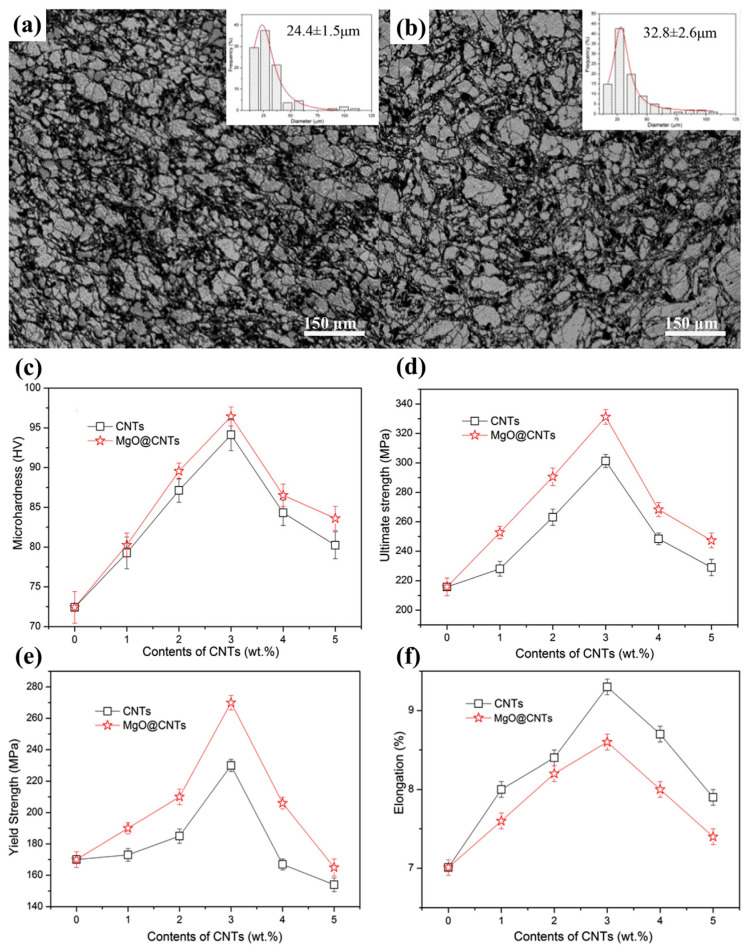
Microstructure and grain size distribution (inset) of AZ91 alloy composites with (**a**) 3.0ewt% MgO @ CNTs and (**b**) 3.0ewt% CNTs; effect of the addition of purified CNTs and MgO @ CNTs on the mechanical properties: (**c**) microhardness, (**d**) ultimate strength, (**e**) yield strength, (**f**) elongation [90].

Yuan et al. [90] synthesized magnesium oxide-coated carbon nanotube (MgO @ CNT)-reinforced AZ91 alloy composites by the powder metallurgy method. In their study, MgO nanoparticles were incorporated on carbon nanotubes to reinforce a semi-coherent MgO/Mg interface. Nano-scale contact interface and diffusion bonding interface were formed between carbon nanotubes and magnesium oxide (Figure 14a,b). The presence of MgO nanoparticles significantly improved the interface bonding strength, thus ensuring the effective transfer of load between the matrix and carbon nanotubes. When the content of MgO @ CNTs was increased to 3.0 wt%, the yield strength and ultimate tensile strength of MgO @ CNT/AZ91 composites were increased to 284 MPa and 331 MPa, respectively, and the elongation was 8.6%, which was 69.0%, 54.0% and 22.9% higher than that of pure AZ91 alloy, respectively (Figure 14c–f).

**Figure 15:**
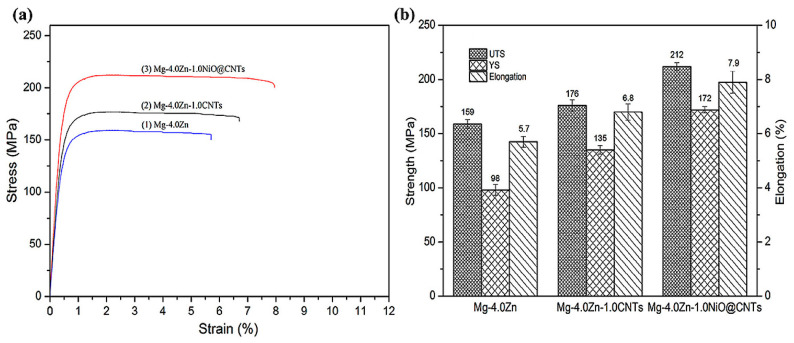
(**a**) Stress–strain curves of different materials; (**b**) mechanical indexes of different strengthening materials [91].

Yuan et al. [91] also synthesized NiO-coated CNT (NiO @ CNTs)-reinforced Mg-4.0Zn composites by ball milling and casting. The results indicate that the interface bonding is improved due to the coating NiO nanolayer on the carbon nanotubes. In addition, the formation of MgO and Mg_2_Ni phases at the CNTs/Mg interface effectively prevent the aggregation of carbon nanotubes. The good dispersion of reinforcements and the improvement in interfacial bonding effectively improve the mechanical properties of the composites. As shown in Figure 15, the mechanical properties of Mg-4.0Zn-1.0NiO @ CNTs composites are obviously better than those of the Mg-4.0Zn alloy and Mg-4.0Zn-1.0CNTs composites. Especially, the yield strength, elongation at break and tensile strength of Mg-4.0Zn-1.0NiO @ CNTs composites are 145 MPa, 7.9% and 212 MPa, respectively, which are 44.9%, 38.6% and 33.3% higher than those of Mg-4.0Zn alloy, respectively. It can be seen that the appropriate surface treatment of the reinforcement improves the interface strength and chemical bonding significantly, which is beneficial to the improvement in the comprehensive mechanical properties of the composite material.

The interfacial bonding strength can be controlled by optimizing and adjusting the preparation process parameters in various manufacturing processes. Temperature is often the factor that has the greatest influence on the reaction rate. Composite materials are generally prepared at high temperature [92]. The activity of many elements will increase at high temperature, resulting in the strong interfacial reaction due to the diffusion of elements [78,81]. Therefore, it is necessary to avoid a too high preparation temperature or long-time heat preservation at a high temperature. Taking the high-pressure casting process as an example, the solidification process of magnesium alloy is usually divided into two stages: the formation and growth of a crystal nucleus [93]. The formation of crystal nucleus is a key process that may affect the stability of the solid/liquid interface. A primary phase with dendritic structure (a-Mg dendrite) is formed due to the existence of disturbance factors such as temperature and composition [94], which has an important influence on the mechanical properties of alloy components [95,96,97]. Therefore, optimizing the process parameters in the preparation of composite materials will also have a beneficial effect on the stability of interface bonding.

## 5. Properties and Applications of Nanoparticle-Reinforced Magnesium Matrix Composites

Nanoparticle-reinforced magnesium matrix composites (MMCs) generally have the characteristics of high specific strength, high specific stiffness, low density, low CTE, high thermal stability, high chemical stability and good high temperature properties (tensile properties, creep resistance, fatigue resistance, etc.). These remarkable properties make it possible for MMCs to become the ideal composite material with integrated structure and function in the transportation, aerospace, biomedicine and automobile fields [81]. For example, it can save energy and reduce emissions in the field of transportation, save weight and reduce carbon dioxide emissions in the field of aerospace, and can be used as artificial bone tissue parts in the field of medicine. Additionally, in the field of military, space and other cutting-edge technologies, it can be used as structural materials or functional materials for manufacturing satellites, high-precision airborne radar antennas, and precision navigation.

### 5.1. Mechanical Properties of Nanoparticle-Reinforced Magnesium Matrix Composites

Low-alloy and RE wrought alloys are the main focus of magnesium alloys because of strength-ductility synergy. Nano-grain strengthening is the primary strengthening mechanism [98]. Jia et al. [99] prepared Mg-12Gd-1Er-1Zn-0.9Zr using hot extrusion pre-deformation and two-stage aging treatment. The YS, UTS and the elongation of the alloy are 506 MPa, 549 MPa, and 8.2%, respectively, which realizes the synergistic improvement in plasticity and strength. High-density dislocations generated during the two-stage aging process cause work hardening and precipitation, resulting in increased strength. In addition, Yu et al. [100] developed a low-Re content Mg-3Y-2Gd-1Nd-0.5Zr (wt%) alloy with high strength and ductility. The results show that the long-range chain structure and the deformed grains in the non-precipitated zone in the low Re containing magnesium alloy can achieve the synergistic improvement in strength and plasticity [101,102,103]. Pan et al. [104] fabricated a Mg-Ca-based alloy with ultra-high strength and plasticity by low temperature extrusion. They found that Ca element can improve the creep resistance and refine the grains. In addition, nanocrystalline phase is produced under low temperature extrusion, so as to realize the synergistic improvement in plasticity and strength.

The low-cost magnesium alloys, as the lightest structural metal material, have been proved to become a promising candidate for structural and engineering applications in the automotive and aerospace industries due to their high strength-ductility synergy. Liu et al. [105] developed a MMCs using SiC powder and wire cold metal transfer (CMT) process. The addition of the SiC nano-reinforced particles of 49.1 nm greatly improves the mechanical properties of the Mg-Al alloy, in which the maximum tensile strength is increased to 325.616 MPa. Meng et al. [55] prepared reinforced magnesium matrix composites with graphene nanosheets (GNPs) by hot pressing and rolling to obtain higher tensile strength than pure magnesium (0.25 vol% GNP composites showed 160 MPa tensile strength). The results indicate that the UTS of the composites increases with increasing GNP content. In addition, the induced layer structure suppresses the dislocation movement and the crack propagation, and keeps the elongation at an objective level. Labib et al. [106] prepared MMCs reinforced by nano-SiC articles with different volume fractions by powder metallurgy technology. The shear yield stress and ultimate shear strength of the composites increase significantly with the increasing dimensional stability. The dimensional stability needs to be strictly controlled in the performance regulation of MMCs. Compared with other ceramic reinforced materials, silicon carbide particles have lower cost and higher thermal properties [107]. Powder metallurgy technology provides cost-effective and high properties at high temperatures compared to conventional manufacturing processes [108]. Tu et al. [109] developed a high modulus and high strength Mg-Gd-Ag-Mn-Ge alloy. The elastic modulus, strength and elongation reached 51 GPa, 423 MPa and 10%, respectively. The formation of a high modulus Gd_5_Ge_3_ phase due to the addition of Ge element caused solid solution hardening and grain boundary strengthening, significantly improving the modulus and strength of the alloy. The incorporation of large modulus elements can effectively regulate the comprehensive properties of MMCs, which greatly improve energy efficiency in the aerospace field and stimulate the potential of lightweight structural systems such as automotive and mobile electronic applications [110].

Particle-reinforced magnesium matrix composites provide a good way to solve the shortcomings of insufficient strength and toughness of magnesium alloys, and have a wide range of applications. However, the problem of coarse particle size is common in such composites, and the substitution of nanoparticles provides a good way to solve this problem, and the magnesium matrix composites prepared by introducing nanoparticles have higher yield strength, tensile strength, plasticity and toughness. The advantages have attracted the research of scholars. In addition, the significant improvement in mechanical properties makes magnesium matrix composites have a wider application prospect in rail transit and aerospace fields.

### 5.2. Hardness and Abrasion Resistance of Nanoparticle-Reinforced Magnesium Matrix Composites

Meher et al. [62] prepared TiB_2_ reinforced magnesium matrix RZ5 alloy matrix composites by self-propagating high temperature synthesis method and conducted wear experiments. The results indicate that the hardness and strength of the composites increased with increasing content of TiB_2_ particles, while the wear rate decreased. Turan et al. [111] prepared fullerene reinforced MMCs by semi-powder semi-metallurgical method and they found that there was an increase in hardness and a decrease in friction. In addition, Turan et al. [112] prepared MMCs reinforced with different graphene contents by the semi-powder semi-metallurgy method. Under the condition of preventing agglomeration of graphene-reinforced particles, the higher the GNP content, the lower the corrosion resistance of the composites. Thrugnanasambandham et al. [59] improved the wear resistance and friction coefficients of magnesium alloy by manufacturing MMCs reinforced with Al_2_O_3_ nanoparticles (50nm) by the stirring casting process. Banerjee et al. [113] studied the microhardness and density of Mg-WC nanocomposites with different contents of tungsten carbide at high temperature and found that the wear and friction performance of Mg alloys were significantly improved. Zhang et al. [114] used carbon nanotubes to strengthen AZ91D MMCs by cyclic extrusion, which refined the matrix particles and improved the hardness, but reduced the elongation at break. Pal et al. [115] fabricated Al-WC nanocomposites by ultrasonic assisted stirring method. The microstructure analysis and microhardness test revealed that the addition of WC particles improved the wear resistance and tribological performance of the composites.

The metal–matrix composites (MMCs) reinforced by nanoparticles such as TiB_2_, Al_2_O_3_, fullerene, GNPs, etc., have received a tremendous attention in automotive and aerospace industries due to their superior specific strength, specific stiffness and low density. For example, the automotive industry focuses on the application of magnesium and its alloys, such as dashboards, steering wheels, engine mounts, seats, and many different housings [116]. Moreover, in the field of electronic industry, the shell material of electronic devices requires high strength, high hardness and good shock absorption. Obviously, traditional plastic and aluminum materials have had difficulty meeting all these requirements. Therefore, MMCs with these properties has broad application prospects in electronics and household appliances. However, with the increase in TiB_2_ content, the brittleness of the material decreases dramatically [62]. Additionally, the inclusion of fullerene to the matrix increases resistance to corrosion in the material.

### 5.3. Corrosion Resistance of Nanoparticle-Reinforced Magnesium Matrix Composites

Jaiswal et al. [117] synthesized Mg-3% Zn matrix composites reinforced with HA by the powder pressing method and the conventional sintering method. The results indicate that the corrosion rate was successfully reduced and the compressive yield strength was improved, resulting in an increase in compressive strength and elastic modulus to a certain degree. Zhang et al. [118] found that the addition of silicon carbide (SiC_p_) nanoparticles increased the physical and mechanical properties of extruded AZ91 MMCs (EX-AZ91/SiC_p_ MMCs), but significantly reduced their corrosion resistance. Xu et al. [119] deeply studied the extrusion, heat treatment and rolling processes of lithium-rich magnesium alloys (realizing solute nanostructures), and found that these processes can enhance resistant to corrosion. Banerjee et al. [68] prepared AZ31 magnesium matrix composites with different weight fractions of tungsten carbide (WC) nanoparticles. The electrochemical tests indicate that the microhardness, surface roughness and corrosion resistance of the composites in 3.5% sodium chloride solution increase with the addition of WC nanoparticles. Especially, Mg nanocomposites containing 0.5 wt% WC are found to have the strongest corrosion resistance. Zhang et al. [120] deposited a calcium stearate-based super-hydrophobic coating on the magnesium substrate after the plasma electrolytic oxidation treatment. Due to the super-hydrophobicity of the coating, the cell compatibility and corrosion resistance of magnesium-based alloys are significantly improved. Zhao et al. [121] studied the microstructure, mechanical properties and biological corrosion properties of extruded Mg-Sr alloy materials by X-ray diffraction, tensile compression test and immersion test. The experimental results show that the tensile strength and compressive strength of Mg-Sr alloy increased with the addition of Sr element. At the same time, Mg-0.5Sr alloy presented the best corrosion resistance and no cytotoxicity.

Ultra-light magnesium alloys show important application prospects in automotive, defense, electronic products, aerospace and biomedical fields, owing to their high strength and high corrosion resistance [119]. For example, the corrosion resistance of MMCs enables them to be used as materials for aircraft propeller casings, and their corrosion resistance provides a strong support for their use as an important transport bearing material in the field of transportation, especially maritime transport. However, the poor corrosion resistance of magnesium alloys limits their further application. Therefore, improving the corrosion resistance of magnesium alloys is extraordinarily critical to prolong service life and expand their applications [122]. In addition, the enhancement of corrosion resistance and the retention of mechanical properties have greatly improved the potential of the materials in orthopedic applications, such as orthopedic accessories that support fractures and damaged bones.

### 5.4. Biological Properties of Nanoparticle-Reinforced Magnesium Matrix Composites

The aim of this section is to discuss the mechanical properties, biodegradability and compatibility of magnesium matrix composites for orthopedic applications. Cui et al. [73] prepared Mg-5.5Zn/HA composites by spark plasma sintering. The results indicate that the compressive YS and flexural strength are continuously increase with increasing HA content; meanwhile, the anti-degradation ability is also improved. In addition, in vitro experiments identify the composites have a good cell compatibility. Therefore, the high specific strength and great biocompatibility make magnesium matrix composites the ideal materials for biomedical metallic implants. The improvement of the anti-degradation ability also makes it retain good mechanical integrity and effectively prevents secondary fractures of bones. In addition, Zn is considered a biodegradable and non-toxic element that participates in the synthesis of various enzymes in the human body and has anti-inflammatory effects [123,124,125]. β-TCP also has emerged as one of the most promising bone repair materials due to its good bone conductivity, biocompatibility, biological activity, biodegradability and biological absorption properties. Pan et al. [74] prepared β-TCP reinforced zinc-magnesium alloy matrix composites by mechanical stirring, ultrasonic-assisted casting and a subsequent hot extrusion process. The results suggest that the corrosion resistance of the material decreases with an increase in β-TCP content, while its yield strength reaches 250.8 MPa, ultimate tensile strength reaches 330.5 MPa, and elongation reaches 11.7%, respectively. In addition, comprehensive cytotoxicity tests and animal experiments show that the composites have better biocompatibility and higher safety than that of the matrix alloy. As one of the essential elements of the human body, zinc plays a vital role in many aspects such as genes, nutrition and immunity [126] due to its good biodegradability, which can stimulate the growth of new bone and can be used as a bone substitute implant [127]. However, the modulus of magnesium is basically close to that of nature bone due to the defects in mechanical properties. With the introduction of metal matrix composites, it can be more widely used as the implant materials [128,129,130].

Magnesium alloy has become one of the new biomaterials because of their similar elastic modulus to cortical bone, which can eliminate the stress shielding in implantation and can also be degraded in vivo and safely absorbed or metabolized by the human body to avoid secondary surgery. Scholars successfully developed a patented medical magnesium alloy JDBM [131,132,133] implant with uniform controllable degradation, strong toughness matching and good biocompatibility by controlling the phase potential and microstructure design of magnesium alloy materials. Subsequently, they developed a patented coating technology with biological activity, which can be degraded by itself and can inhibit the excessive degradation of the matrix. The technology successfully solved the adverse phenomenon of hydrogen accumulation caused by the excessive degradation of magnesium alloy materials in the body [134,135,136]. By synergistically regulating the degradation behavior of the material matrix and functional coating, the uniform and controllable degradation of medical magnesium alloy intraosseous implant devices has been realized. Xie et al. [137] applied a biodegradable magnesium alloy to surgeries, and no complications such as infection, internal fixation failure, or malunion were observed after fracture healing. The study confirmed the clinical efficacy and biosafety of degradable magnesium alloy screws in the treatment of medial malleolus fractures. The research laid a solid foundation for the further clinical application of high-end medical devices such as fully degradable magnesium alloy implants.

In summary, there are numerous research applications and development opportunities for magnesium matrix composites in the realm of biomedical materials. We anticipate using magnesium matrix composites in bone repairs, cardiovascular interventional therapy, nerve repairs, and tissue engineering through the integration of medicine and engineering, owing to their strong biocompatibility and biodegradability. In addition, further study is required to address the issues of biodegradation rate, the impact of corrosion products on microstructure, mechanical characteristics, and processing qualities in order to facilitate the widespread use of magnesium matrix composites in the field of biomedical materials.

### 5.5. Creep Behavior of Nanoparticle-Reinforced Magnesium Matrix Composites

Zhang et al. [138] investigated creep behavior and microstructure evolution of cast Mg-48m-2Yb-0.6Zn-0.4Zr alloy, and they found that the creep activation energy ranged from 112 to 504 KJ/mol. Cross-slip and climb of dislocations were the primary creep mechanisms, according to change of the dislocation substructure. In the alpha-Mg matrix, the precipitation of prismatic beta phases and basal gamma phases during creep formed created closed volumes, effectively stopped dislocations and contributing to the higher creep resistance of α-Mg matrix that of most creep-resistant magnesium alloys, including die-cast magnesium benchmark alloys and conventional cast magnesium alloys based on Mg-RE. Zhang et al. [139] prepared a typical Mg-13Gd-6Y-0.2Zr (wt%) by combining isothermal forging and artificial aging. They found that the forging exhibited exceptional creep resistance with a steady-state creep rate of 2.26 × 10^−9^/s at 200 degrees C and a tension of 60 MPa. The recrystallization during isothermal forging refined the grains and boosted strength. The consistently distributed beta phase (30–50 nm) formed during aging contributed to the effective strengthening. Bai et al. [140] prepared the new high pressure die casting (HPDC) Mg-RE-based alloy with excellent heat resistance and creep resistance. Its tensile strength at 200 °C reached 229 MPa and the minimum creep rate at 200 °C/70 MPa was 1.76 × 10^‒10^ s^−1^. The fine grain structure produced by the co-segregation of Y and Zn atoms and the coherent long period stacking order (LPSO) [141] with network distribution are beneficial to obtain excellent heat resistance and creep resistance. Qin et al. [142] studied the tensile creep behavior of Mg-10Gd-0.4Zr alloy after heat treatment at 225~300 °C and in the load of 50~80 MPa. The results demonstrated that the critical transition temperature had an impact on the creep behaviors of solution-treated (T4) and peak-aged (T6) alloys. Microstructure evolution showed that the creep resistance of T6 alloy was better than that of T4 alloy below the critical transition temperature due to the presence of additional beta phases. In addition, the performance of magnesium matrix composites fabricated by additive manufacturing is further improved [143]. For example, nano-porous metals manufactured by additive manufacturing have excellent mechanical properties and biocompatibility, and are mostly used for load-bearing orthopedic and dental implants with high requirements for light weight and high strength [144].

It is not difficult to find that the nanoparticle-reinforced magnesium matrix composites can effectively improve the high temperature creep resistance of the magnesium alloy from the two aspects of composition design and microstructure regulation of micro-rare earth alloy. Magnesium matrix composites have great potential to become high-performance heat-resistant alloys. In addition, the excellent creep resistance of magnesium matrix composites in high temperature environments makes them have great potential for applications in the rail transit, aerospace and national defense industries. At the same time, as the lightest metal structural material currently used, magnesium alloy has been paid more and more attention in the fields of new energy vehicles and aerospace, and has been more widely used, playing an irreplaceable role in lightweight, energy saving and emission reduction.

## 6. Conclusions

Nanoparticle-reinforced magnesium matrix composites make up for the defects of traditional magnesium alloys with high weight and cost and general mechanical properties [5,8,145]. This paper focuses on improving the mechanical properties of magnesium matrix composites, such as hardness, strength and wear resistance by the addition of ceramic enhancers [146]. Different magnesium matrix composites formed by the addition of Li, Al, Zn and rare earth elements to magnesium alloys can significantly enhance the mechanical properties of magnesium matrix composites. Among them, the Mg-RE matrix composite prepared by the in situ synthesis method has a great potential for future application as the structure materials due to their excellent mechanical properties. The strength of magnesium alloy can be greatly improved by adding rare earth RE element [4].

The interfacial bonding between reinforcement and matrix plays a crucial role to the final properties of magnesium matrix composites. In order to improve the mechanical properties of magnesium matrix composites, a good reinforcement with superior mechanical properties should be added to minimize the interfacial reaction between the magnesium matrix and the reinforcement, and then effectively transfer the pressure acting on the matrix to the reinforcement. In recent years, various metal-reinforced magnesium matrix composites have been developed, and the yield strength, the ultimate tensile strength and the elongation were improved [44]. By comparison, ceramic particle- and carbon-reinforced magnesium matrix composites are more prone to agglomeration, which limits the continuous and uniform distribution of reinforced materials. Additionally, the poor wettability between the reinforcement and the magnesium matrix can cause a decrease in the interfacial bonding strength. In recent years, many researchers have added various ceramic reinforcements to magnesium matrix, which greatly enhanced the mechanical properties of the materials. Moreover, magnesium matrix composites have a wide range of applications in the biomedical field because of their properties such as high strength, low density and degradability.

Enhancing the interfacial bonding strength is considered an essential approach to enhance the mechanical performances of magnesium matrix composites. Good wettability and appropriate interfacial reaction effectively increase the strength of the interfacial bond. The in situ synthesis used to prepare magnesium matrix composites has the advantages of reducing the intensity of interface reaction and improving wettability, which can effectively improve the interfacial performance [42]. In addition, preparing a specific nano-metal or nano-metal oxide coating on the surface of the reinforcement to form a new phase at the interface is regarded as a unique approach to uniformly disperse nanoparticles to optimize their mechanical performances. Temperature, as an important process parameter affecting the interface state, also plays an important role in deciding the final properties of magnesium matrix composites. Therefore, an appropriate temperature and holding time at high temperature should be used during the processing.

Magnesium matrix composites have potential uses in the automotive industry and aerospace industry due to their low density, wear resistance, high strength and ductility [147]. In addition, clinical trials have confirmed the biosafety and clinical efficacy of magnesium matrix composites to a certain extent as an excellent biomaterial. In order to achieve comprehensive development and further industrial application of nano-reinforced magnesium matrix composites, the following aspects still need to be studied. Firstly, the optimal combination of matrix alloy composition should be further explored for specific properties. Rare earth elements have been proved to cooperate with reinforcements in the magnesium matrix to improve thermal conductivity. So far, the strengthening mechanism of rare earth on the composites is still unclear. Secondly, magnesium-based hybrid composites should be further studied. The addition of multiple reinforcing materials can make up for the performance defects of a single reinforcing material due to combining the advantages of each reinforcing material. At the same time, magnesium hybrid composites also have a good wettability of the reinforcement and facilitate large-scale production [4]. Finally, an appropriate interfacial bonding strength must be obtained to achieve the best strengthening and toughening effect of magnesium matrix composites. As a widely used preparation method, the in situ synthesis method can effectively synthesize magnesium matrix composites with high interfacial bonding strength. In addition, the formation of effective surface coatings can prevent the infiltration of matrix ions and prevent interface reactions to a certain extent [42]. Exploring the mechanism of interface reaction and control are the future research directions for the development of nano-particles reinforced magnesium matrix composites.

## Figures and Tables

**Table 1:** The comprehensive features of nano-reinforced MMCs are reviewed.

Matrix in MMCs	Defects (Not in MMCs)	Special Properties (IN MMCs)	Refs.
AZ (Mg-Al-Zn)	(1) Low corrosion resisting (2) Low strength and hardness	(1) Excellent electromagnetic interference shielding performance (2) Preferable YS and UTS (3) Wear resistance (4) Used for additive manufacturing	[26,27]
WE (Mg-RE)	(1) Expensive (2) Rare earth purification	(1) Corrosion resistance (2) Creep resistance (3) Relatively cheap (compared to multi-component alloys)	[4,28,29]
Mg-Li	(1) Low modulus (2) Low strength and hardness	(1) Light weight (2) Specific modulus (3) Light while possessing high strength	[32,33,34]
Mg-Zr	Corrosion resistance	(1) Biocompatibility (2) Corrosion resistance (3) Non-toxic	[36]

**Table 2:** Summary of mechanical properties of some typical metal reinforced MMCs.

Alloy	Reinforcement	TS (MPa)	YS (MPa)	UCS (MPa)	EL (%)	Second-Phase	Refs.
AZ31	15 vol% Fe–18Cr–9Ni	355	241	—	13	—	[53]
	15 vol% Ti–6Al–4V	340	220	—	12	—	[53]
	15 vol% Al–5Mg–3Zn	280	175	—	8	—	[53]
	0.5 wt% 3Al-Fe	338	282	—	13.8	Mg_17_Al_12_, Al_3_Fe	[52]
	Ti-Ni	269	140	—	—	Al_3_Ni_2_	[49]
AZ91	5 wt% Ti-6Al-4V	303	211	—	18.7	Al_3_Ti, Mg_21_(Zn, Al)_17_	[47]
	10 wt% 0.5Al-Co-Cr-Fe-2Ni	—	—	209 ± 8	—	Mg_17_Al_12_	[51]
Mg-15Al-6Zn-2Cu	3 wt% Ni	—	—	403.7	—	TiNi	[50]
Mg–3Al–1Zn	9 wt% Ti	294	264	—	8	TiAl	[48]

**Table 3:** Properties of common ceramic reinforcements.

		Density (g/cm^3^)	Melting Point (°C)	Crystal Structure	Elastic Modulus (GPa)	Thermal Conductivity (W/mK)	CET (μ/K)	Refs.
oxide	Al_2_O_3_	3.97	2054	Hexagonal	400	30	7–8	[47,48]
	Y_2_O_3_	5.01	2410	Cubic	—	27	8	[48]
carbide	SiC	3.22	2700	Hexagonal	400	40–60	5.12	[45,52]
	WC	15.63	2870	—	630	85	6.9	[46,49]
nitride	AlN	3.26	2249	Hexagonal	314	285	4.5	[44,45]
	BN	2.2	3000	Hexagonal	90	25	3.8	[42,48]
boride	TiB_2_	4.52	3225	Hexagonal	565	60–120	8.1	[4,48]
	ZrB_2_	6.09	3000	Hexagonal	350	23	5.9	[45,49]

**Table 4:** Compression properties of AZ91 alloy and AlN/AZ91 composites with different nitridation reaction times [67].

Nitridation Reaction Times (h)	Yield Strength (MPa)	Ultimate Compressive Strength (MPa)	Compressive Fraction Elongation (%)
0	93 ± 4	313 ± 10	18.1 ± 1.5
1	100 ± 2	347 ± 4	19.6 ± 0.4
1.5	108 ± 1	360 ± 5	20.3 ± 0.5
2	110 ± 2	371 ± 8	22.1 ± 0.4
2.5	95 ± 5	333 ± 13	20 ± 1.1

## Data Availability

Not applicable.
